# Chemical Classes Presenting Novel Antituberculosis Agents Currently in Different Phases of Drug Development: A 2010–2020 Review

**DOI:** 10.3390/ph14050461

**Published:** 2021-05-13

**Authors:** Klaudia T. Angula, Lesetja J. Legoabe, Richard M. Beteck

**Affiliations:** Centre of Excellence for Pharmaceutical Sciences (Pharmacen), North-West University, Potchefstroom 2520, South Africa; talohole3@gmail.com (K.T.A.); lesetja.legoabe@nwu.ac.za (L.J.L.)

**Keywords:** tuberculosis, drug development, pharmacokinetics, fluoroquinolones, diarylquinolines, nitroimidazoles

## Abstract

Tuberculosis (TB), caused by *Mycobacterium tuberculosis* (Mtb), is a curable airborne disease currently treated using a drug regimen consisting of four drugs. Global TB control has been a persistent challenge for many decades due to the emergence of drug-resistant Mtb strains. The duration and complexity of TB treatment are the main issues leading to treatment failures. Other challenges faced by currently deployed TB regimens include drug-drug interactions, miss-matched pharmacokinetics parameters of drugs in a regimen, and lack of activity against slow replicating sub-population. These challenges underpin the continuous search for novel TB drugs and treatment regimens. This review summarizes new TB drugs/drug candidates under development with emphasis on their chemical classes, biological targets, mode of resistance generation, and pharmacokinetic properties. As effective TB treatment requires a combination of drugs, the issue of drug-drug interaction is, therefore, of great concern; herein, we have compiled drug-drug interaction reports, as well as efficacy reports for drug combinations studies involving antitubercular agents in clinical development.

## 1. Introduction

Tuberculosis (TB) is a contagious bacterial infection—one of the top ten causes of death, especially in young adults worldwide [1]. TB is the leading cause of mortality from a single infectious agent, ranking above human immunodeficiency virus (HIV) and malaria [2]. *Mycobacterium tuberculosis* (Mtb), a bacterium discovered by Dr. Robert Koch in 1882, is the causative agent of TB, and it is a member of the *Mycobacterium tuberculosis* complex (MTBC). MTBC is composed of several subspecies responsible for TB in mammals [3]; while all these subspecies were isolated, only five of them are known to commonly cause TB in humans [4]. The primary organs of Mtb’s aerobic infection are the lungs, resulting in pulmonary TB. From the lungs, the infection can spread to other organs (brain, bone marrow, and spine), leading to extrapulmonary TB [1]. The common mode of TB transmission is through droplet nuclei from an infected person to others, which are generated during coughing, sneezing, or speaking [5].

Although TB affects both HIV-positive and HIV-negative people, the number of deaths estimated in HIV-negative people outweighs the former. There were an estimated 1.3 million TB-related deaths among HIV-negative people and 300,000 deaths among HIV-positive people in 2018 [2]. This amounts to nearly 2 million TB-related deaths every year, a number that has been relatively stable in recent years [2]. Co-infection with HIV infection greatly increases the chances of an individual developing active TB following exposure, and also immunosuppression related to uncontrolled HIV infection leads to reactivation of latent TB [6]. Additionally, Smoking, diabetes, and malnutrition increase susceptibility to active TB; silicosis, organ transplant, tumor necrosis factor-alpha inhibitors are other risk factors for reactivation of latent TB [7].

In addition to the above-mentioned risk factors, antimicrobial resistance (AMR), mainly the multidrug-resistant Mtb (MDR-Mtb), extensively drug-resistant Mtb (XDR-Mtb), and totally drug-resistant Mtb strains (TDR-Mtb) are also implicated in the increasing TB prevalence to an extend [8]. The problem of MDR-TB and XDR-TB across the world has become very alarming as a direct consequence of mistakes and adherence to the prescribed chemotherapy, availability of anti-TB drugs, surveillance of the patients, and incorrect administration of anti-TB drugs [9]. Other factors contributing to the rise in TB cases include increased immigration from TB endemic countries to countries with low TB prevalence [10] as well as the growing level of homelessness coupled with drug abuse [11]. 

Presently, treatment of drug-sensitive (DS) TB is divided into a two-month intensive phase of treatment comprising of rifampicin, isoniazid, pyrazinamide, and ethambutol (first-line drugs) followed by a four-month continuation phase consisting of rifampicin and isoniazid [12]. Failure of first-line drugs is attributed to several reasons, including non-compliance of patients (affecting clinical and a microbiological cure) [13] and side effects of prescribed drugs [14]. This failure often leads to the emergence of MDR-TB, defined as resistance to isoniazid and rifampicin, the two most potent first-line drugs in the TB treatment regimen [15]. Consequently, the need arises to treat MDR-TB with relatively expensive and more toxic second-line drugs. Moreover, treatment of MDR-TB often requires a longer treatment period (minimum of up to 18 months) [4,7]. The current second-line drugs are grouped into three groups: A, B, and C, according to their efficacy profiles [16]. Group A drugs include the fluoroquinolones (Levofloxacin or moxifloxacin), bedaquiline (BDQ), and linezolid; group B includes clofazimine, and cycloserine or terizidone; group C contains ethambutol, delamanid, pyrazinamide, imipenem-cilastatin or meropenem, amikacin or streptomycin, ethionamide or prothionamide, and *p*-aminosalicylic acid [17,18].

Resistance of Mtb to any fluoroquinolone and to at least one of the injectable drugs (capreomycin, kanamycin, or amikacin), in addition to isoniazid and rifampicin resistance, is termed XDR-TB [15]. Notably, co-infection of HIV and XDR-TB strains are increasingly associated with poor treatment outcomes in HIV-seropositive persons [19].

**Scheme 1:**
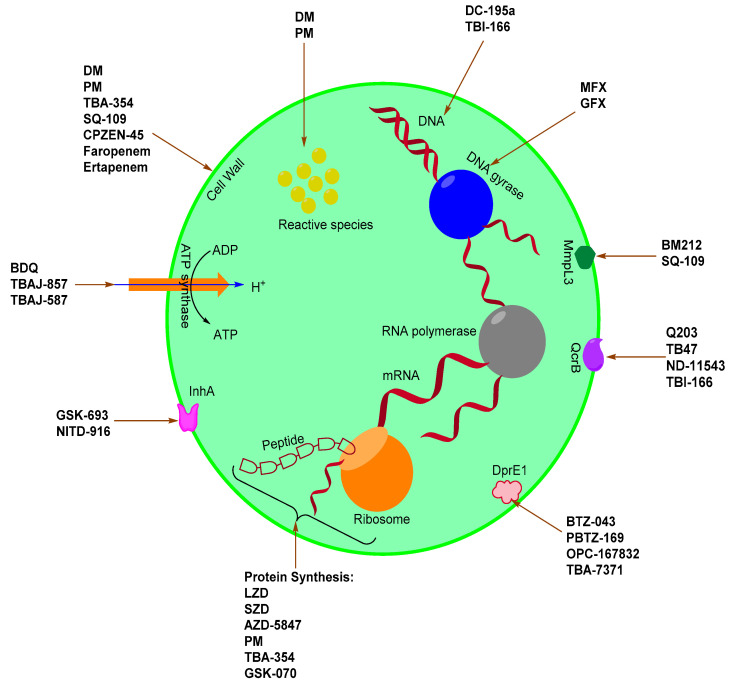
TB drugs and drug candidates and their associated targets.

The compliance and toxicity issues associated with first-line drugs, the emergence and worldwide spread of multidrug-resistant Mtb, the high failure rate of drug candidates in the development pipeline, and the general propensity of bacteria to develop resistance against newly approved drugs make paramount the continuous search of novel compounds with antitubercular activity. To promote the discovery of new antitubercular agents, we herein compiled the different classes of drug candidates in lead optimization, pre-clinical, clinical development, and recently approved drugs, as well as their putative targets (see Scheme 1 below). We search articles from the internet using keywords, such as new TB agents, TB drug pipeline, TB drug candidates, and pharmacokinetic of TB drug candidates. We also consulted sites such as the Working Group on New TB drugs, TB Alliance. New or repurposed compounds under investigation from 2010 onward were also selected. 

## 2. Novel Anti-Mtb Agents

Mtb has a well-developed cell wall which provides an extraordinary lipid barrier. This barrier facilitates the manifestation of either acquired or intrinsic resistance to antibiotics [20]. It is imperative to understand the mode of action of current drugs as well as the mechanisms of bacterial resistance in the pursuit to discover or develop new anti-TB drugs or regimens aimed at combating difficult-to-treat bacterial strains (MDR and XDR) [20,21]. The mode of action for existing and emerging drugs against Mtb include inhibition of the cell wall, cell wall acids, and peptidoglycan (WecA) synthesis, DNA gyrase and topoisomerases, DNA replication, protein synthesis, ATP synthase, Lipid synthesis, DprE1, InhA, QcrB, LeuRS, MmpL3 protein, and *L*,*D*-transpeptidase. This review will discuss in detail most of the drug candidates under respective chemical classes using the stated mechanisms and/or novel modes of action displaying potential for TB therapy evident from activity in pre-clinical and/or clinical trials.

### 2.1. Quinolone Derivatives

#### Fluoroquinolones

Fluoroquinolones generally target the DNA gyrase enzyme in Mtb, which is encoded by both gyrA and gyrB genes [22]. DNA gyrase is a tetrameric A2B2 protein that is unique in catalyzing the relaxation of negative supercoiling of DNA and is essential for efficient DNA replication, transcription, and recombination [23]. The A subunit (90 to 100 kDa) carries the breakage-reunion active site, whereas the B subunit (70 to 90 kDa) promotes ATP hydrolysis, which is needed for energy transduction [24]. Mtb genes encoding DNA gyrase were identified from the genome analysis as a gyrB-gyrA contig in which gyrA and gyrB encode the A and B subunits, respectively [24]. Mutations in the gyrA and/or gyrB subunit of DNA gyrase often lead to fluoroquinolone resistance [25]. Currently, clinically important anti-Mtb fluoroquinolones are the fourth generation fluoroquinolones (moxifloxacin and gatifloxacin) and DC-159a.

(i) Moxifloxacin

**Figure 1:**
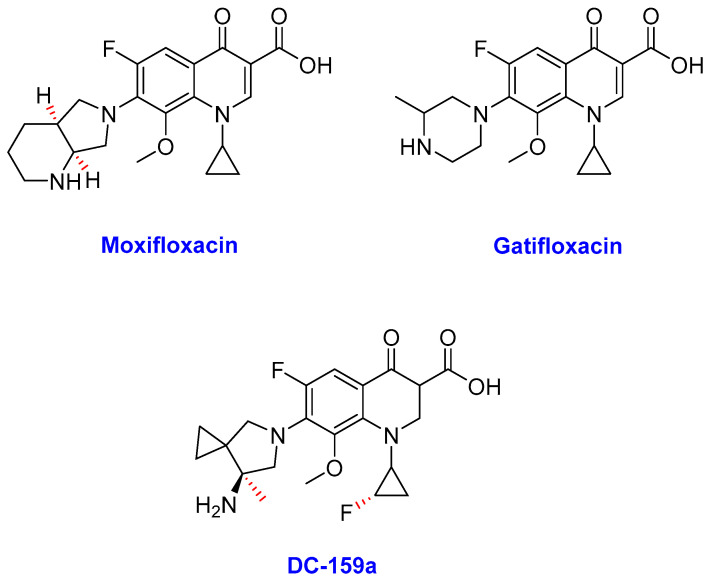
The chemical structures of potential anti-TB fluoroquinolones.

Moxifloxacin (MFX) is an 8-methoxy fluoroquinolone with enhanced activity against wild-type Mtb compared to other fluoroquinolones such as ofloxacin and ciprofloxacin [26]. It is currently approved as an optimum drug for MDR-TB and as a substitute for levofloxacin in the MDR-TB regimen [27]. Moreover, MFX is an alternative drug for patients who are unable to tolerate the standard DS-TB drug regimen [28] and also for XDR-TB patients, provided it exhibits a minimum inhibitory concentration (MIC) of <2 mg/L against the isolate [29]. The structural difference between MFX and other fluoroquinolones (Figure 1), which includes a methoxy group in the C-8 position and the aza-bicyclic group at the C-7 position, contributes to its bactericidal activity, lower MICs, and lower propensity for Mtb to develop resistance against the drug [29]. Therefore, MFX is amongst a small number of highly bactericidal anti-TB drugs and contributes to the development of more effective treatment regimens. This is due to its excellent in vitro and in vivo bactericidal activity [30,31].

MFX is readily absorbed from the gastrointestinal tract following oral administration. After the recommended oral dose of 400 mg, a mean maximum plasma concentration (Cmax) of 3.4 mg/L was reached in 1.5 h (Tmax), and its area under the curve (AUC) was 30.2 mg·h/L, while an equal dose reached a Cmax of 3.62 mg/L and AUC of 34.6 mg·h/L via intravenous administration [26,32]. Noteworthy, co-administration of MFX and rifampicin reduces the Cmax of the latter, and this must be considered in multidrug TB regimens that contain rifampicin [33]. The distribution of MFX is parallel throughout the body, with tissue concentrations often exceeding plasma concentration [34]. Similarly, its rate of elimination from tissues and plasma is generally parallel [35].

As a repurposed drug with existing extensive safety data which indicate good drug-like properties, this drug is expected to have a good safety profile in TB treatment as well [36]. However, it was recognized that MFX used is associated with the risk of QT prolongation, and this risk increases when MFX is used in combination with drugs such as bedaquiline and delamanid [28]; hence careful safety monitoring is employed in these cases.

(ii) Gatifloxacin

Another novel fluoroquinolone undergoing advanced clinical trial is gatifloxacin (GFX), a structural analog of MFX currently in phase III clinical trial. Although GFX is recommended in the WHO guidelines, the need for treatment optimization is very critical for MDR-TB [37]. Grasela (2000) demonstrated that oral and intravenous administration of GFX produces equal dose-dependent Cmax, AUC, and clinical effects on participants regardless of gender or ethnicity. This is comparable to findings from two independent studies consisting of healthy white and Japanese male subjects. These studies showed that both study groups reached similar Tmax (1–2 h) and Cmax (3.8 mg/mL) from a 400 mg dose administered either orally or intravenously [38,39]. Another study optimizing pharmacokinetic/pharmacodynamic parameters of GFX in pulmonary and meningeal MDR-TB established that at 400 mg, GFX has dose-dependent MICs values in the range of 0.5–2 mg/L, while Cmax and AUC_0–24_ are 4.2 ± 1.9 mg/L and 51.3 ± 20.4 mg.h/L, respectively [40]. Like other fluoroquinolones, GFX has high oral bioavailability, and it is well distributed in tissues, wherein it often achieves concentrations that exceed serum concentrations [41]. The drug has a mean concentration in most tissues, including bronchial mucosa, lung epithelial lining, and sinus mucosa, at 1.5–2.0 fold the mean serum concentration [40,42]. Metabolically, GFX is a stable compound with a half-life ranging from 7 to 14 h, and >80% of the drug is excreted through urine unchanged [43].

The potential toxicities widely associated with GFX are dysglycaemia in diabetic and elderly individuals [33]. However, GFX is not significantly associated with clinical drug-drug interactions related to the cytochrome P450 (CYP) enzyme system as opposed to the main drug substituents in the standard DS-TB regimens such as rifampicin and isoniazid; this makes it ideal for co-administration with other drugs [44]. GFX was hailed for good efficacy in a short-term MDR-TB regimen pilot study in Bangladesh [45].

(iii) DC-159a

DC-159a is the newest member of the 8-methoxy-fluoroquinolones with a history of broad-spectrum activities, and its potential use in TB patients has recently been investigated in pre-clinical studies [46]. Analogous to other fluoroquinolones, it acts by inhibiting altered DNA gyrases with substitution(s) at Ala90Val and/or Asp94Gly in GyrA, as well as inhibition of DNA replication of wild-type Mtb [47,48].

In addition to that, DC-159a demonstrates higher potency than MXF and GFX against DS-Mtb, and it retains activity against 11 strains of fluoroquinolone-resistant MDR-Mtb [49,50]. It has an MIC_90_ value of 0.06 µg/mL against DS-Mtb, which is 4 and 8 fold lower than that of the MFX and levofloxacin, respectively [51]. In addition, DC-159a was found to exhibit the MIC_90_ of 0.5 µg/mL against the clinical MDR-Mtb isolates, which are resistant to other fluoroquinolones; for example, MFX and levofloxacin have higher MIC_90_ values of 4 and 16 µg/mL, respectively against the said strain [49]. Its activity is dose-dependent, and the early bactericidal activity (EBA) of DC-159a indicates that it may be a candidate for shortening TB treatment [46]. In a murine TB model study, the dose-ranging activity of DC-159a was compared with that of MXF during the continuation phase as well as the initial phase of treatment. Both MXF and DC-159a exhibited dose-dependent bactericidal activity, with the former displaying superior activity over the latter. Thus, the Cmax for the DC-159a and MXF obtained in mice following single-dose oral administration were 10.28 and 12.46 g/mL, while the AUC_0–__∞_ was 15.80 and 14.69 mg·h/L at 100 mg/kg, respectively [47]. The half-life of DC-159a after a 100 mg/kg administration was observed at 1.51 h and 1.93 h in serum and lungs, respectively [52]. 

The safety profile for DC-159a is not well established, although a monkey model study found the compound to have no effect on CYP 3A4 enzyme [50], phototoxicity, nor chondro-toxicity [53]. However, further evaluation to support substantiation of DC-159a as a novel agent for DS-TB, MDR-TB, XDR-TB, and TB/HIV coinfection cases, as well as further evaluation of intracellular pharmacokinetics and drug-drug interactions are required.

### 2.2. Diarylquinolines

(i) Bedaquiline

Bedaquiline (BDQ) was the first new TB drug from a novel class, which received conditional approval from the US Food and Drug Administration (FDA) in 2012, since rifampicin in 1971 [54]. The novelty of BDQ is unique because, unlike fluoroquinolones, BDQ is a narrow-spectrum antibiotic, exhibiting little activity beyond the mycobacterial species. Notably, BDQ exhibits dual mechanisms of action that specifically target ATP synthase by inhibiting the proton pumping mechanism through binding to the oligomeric (c) and proteolipid (ɛ) subunits of ATP synthase [22,55,56]. These result in bactericidal effects on both active and non-replicating bacilli [57]. Additionally, due to high lipophilicity, which is required by protonophores to move through lipid-rich membranes, BDQ was proposed to possess proton translocation effects associated with electron transport activity in ATP synthesis [58]. BDQ is a member of the diarylquinoline chemical class with a quinolinyl nucleus, an alcohol, and diaryl and amine side chains that are responsible for its antimycobacterial activity; and it is an enantiopure compound with two chiral centers (1R,2S isomer) [59].

Early clinical trials indicated that BDQ is safe and effective in increasing sputum culture conversion and reducing the treatment time required for sputum-negative conversion, making it an effective addition to an MDR-TB regimen [60]. BDQ shows excellent activity against DS-Mtb, MDR-Mtb and XDR-Mtb isolates, with MIC values in the range of 0.03–0.12 μg/mL and shows no cross-resistance with current first-line drugs [61]; this suggests that BDQ retains activity against MDR-Mtb strains [62]. In addition, a once-daily oral administration of BDQ has bactericidal activity at a dose of 400 mg when administered as monotherapy for 7 days in patients with pulmonary TB [63]. Moreover, murine TB model studies indicated that BDQ has potent in vivo bactericidal activity [63]; and it demonstrates synergistic effects with other drugs such as pyrazinamide; thus, BDQ enhances the antibacterial activity of first-line drugs [64]. This may help shorten treatment regimens for DS- and MDR-TB.

BDQ is well absorbed in humans following oral administration, reaching its Cmax in 4–6 h (Tmax) irrespective of the dose [65]. Andries et al. (2005) established that, after administration of the recommended dose of 400 mg/day, BDQ shows a Cmax of 5.5 mg/L and an AUC_0–24_ of 65 mg.h/L; hence the drug clearance (Cl) is around 6.2 L/h [64]. Of interest is the fact that concomitant food intake increases the oral bioavailability of BDQ in healthy subjects. A study on healthy subjects determined that the mean AUC of BDQ administered in a tablet formulation increased by 2.0–2.4-fold relative to fasted conditions [65]. In contrast to other anti-TB drugs, BDQ’s fecal elimination profile is associated with an exceedingly long terminal half-life of 5.5 months, owing to a combination of a long plasma half-life (24 h), high tissue penetration, and long half-life in tissues [64]. Consequently, the emergence of BDQ resistance due to long exposure of Mtb to sub-therapeutic levels of BDQ could be introduced. Therefore, BDQ is suitable for intermittent drug administration.

As a CYP3A4 substrate and due to its high lipophilicity, BDQ causes moderate to high-risk drug-drug interactions with CYP3A4 inducers or inhibitors [66]; thus, affecting its pharmacokinetics. Therefore, co-administration of BDQ with rifamycin’s, such as rifampicin, rifapentine, and rifabutin lead to reduced AUC and Cmax. Rifampicin and rifapentine are both potent CYP isoenzyme inducers, including CYP3A4, while rifabutin causes moderate induction of the same isoenzymes. For example, a study in healthy subjects demonstrated a reduction in the AUC and Cmax of BDQ by up to 59% and 40%, respectively, when BDQ was co-administered with rifampicin [65]. A similar study by the same team confirmed these findings with a different rifamycin, where co-administration of rifapentine with BDQ resulted in 57% and 38% reduction in AUC and Cmax, respectively. On the other hand, rifabutin, as a moderate CYP3A4 inducer, showed a modest reduction in BDQ exposure (10–20%) for both Cmax and AUC [67]. Noteworthy, these activities can be reversed by co-administration of CYP3A4 enzyme inhibitors, such as protease inhibitors, macrolide antibiotics, and azole antifungals [68]. These studies indicate that the metabolism of BDQ is enhanced by co-administration with rifamycins; hence the administration of these drug combinations is restricted to the treatment of DS-Mtb.

In contrast, co-administration of BDQ and some antiretrovirals (ARVs), such as lopinavir which is both a substrate and inhibitor of CYP3A4 isoenzymes (given as a drug combination: lopinavir/ritonavir), leads to a 22% increase in BDQ’s AUC while Cmax is unaffected [69]. Although this is consistent with the effect of CYP3A4 metabolism on BDQ, this combination has a different effect on the BDQ metabolite, where the Cmax and AUC are decreased by 51% and 41%, respectively [69]. In addition, the mean pre-dose concentrations of lopinavir and ritonavir decreased with BDQ co-administration by 21% and 14%, respectively, suggesting that BDQ may have an effect on the pharmacokinetics of lopinavir/ritonavir through an unknown mechanism [65]. Furthermore, upon co-administration with efavirenz, the AUC of BDQ decreased by 18% while its Cmax was not affected [60]. Contrary to this, the BDQ metabolite demonstrated an exorbitant rise in the Cmax (89%), while its AUC was unaffected. This suggests that efavirenz could reduce exposure of BDQ and its metabolite by up to 50% upon chronic co-administration, and therefore their combination, or other CYP3A inducers, should be avoided [70]. Co-administration of BDQ with other drugs such as nevirapine as well as the drugs in the standard MDR-TB regimen (pyrazinamide, ethambutol, kanamycin, ofloxacin, and cycloserine) have negligible effects on the pharmacokinetics of BDQ and vice versa [71].

Moreover, BDQ has pronounced safety issues, including hERG (human Ether-à-go-go-Related Gene) toxicity as well as ADME (absorption, distribution, metabolism, and excretion) issues due to its high lipophilicity [72]. Furthermore, co-administration of BDQ with drugs associated with potential QT-prolongation, including fluoroquinolones (especially MFX), delamanid (DM), macrolides, clofazimine (CFZ), is further cautioned [57]. BDQ has a “black-box” warning for potential induction of long QT syndrome, which can lead to abnormal and potentially fatal heart rhythm [73]. Apart from an unexplained increase in the risk of mortality, BDQ may be associated with nausea, arthralgia, headache, and liver injury [54,57]. Therefore, the WHO places caution on the potentially toxic effects of BDQ that originated from the observation of ten unexpected late deaths in a phase IIb trial study [67]. Consequently, BDQ is undergoing further Phase IIb and III clinical development for MDR-TB indications, although the WHO recommended its application under the group D2 treatment category [22].

(ii) TBAJ-876

Although BDQ is a highly efficacious drug, the pharmacokinetics and toxicological liabilities coupled with its application in MDR-TB treatment inspired further research interests, which consequently led to the discovery of second-generation BDQ analogs; thus, TBAJ-876 is amongst the recent discoveries of 3,5-dialkoxypyridine analogs of BDQ possessing higher potency, low lipophilicity, higher Cl and lower cardiotoxicity [74,75]. The compound has undergone several in vitro and in vivo studies and obtained results that qualified it for pre-clinical studies. As a result, TBAJ-876 is currently investigated further in advanced pre-clinical studies through SAR of its metabolites in order to establish the drug’s impact on safety and efficacy [76]. The lower lipophilicity observed in TBAJ-876 as compared to BDQ is a result of the structural differences between the two compounds. The naphthalene and phenyl moieties present in BDQ are replaced by the more hydrophilic 2,3,5-trialkoxypyridin-4-yl and 3,5-dialkoxypyridin-4-yl moieties, while the quinoline, dimethylamino, and hydroxyl moieties are retained in the TBAJ-876 [77]. Biochemical, genetic, and biophysical analyses suggest that TBAJ-876 inhibits the mycobacterial F-ATP synthase similarly to BDQ by a dual mechanism—interfering with the functions of both the enzyme’s c and ɛ subunits [74]. This supports an on-target mechanism of action model in which targeting both components of the enzyme is required for effective inhibition of Mtb enzyme activity.

In a murine model of TB, TBAJ-876 demonstrated activity similar to BDQ at the same dose, with a lower calculated lipophilicity (clogP) of 5.15, higher IC_50_ value against hERG (>30 µM), and, in most cases, higher Cl values in human microsome (2 µM/min/mg protein) [78]. In addition, the study observed an MIC_90_ value of 0.006 µM and the AUC_0–__∞_ of 4.61 µg.h/mL for TBAJ-876, which was 4-fold that of BDQ while there was no difference in colony-forming units (CFUs) between the two compounds (i.e., >5.5 log_10_ CFU) [79]. Subsequently, advanced studies that evaluated the PK of TBAJ-876 and TBAJ-587 (discussed below) against BDQ demonstrated lower MICs in clinical isolates of Mtb, efficacy against murine TB at lower exposures, lower potency against hERG, predicted higher human Cl, and an acceptable safety margin, based on the safe exposure in rats [78].

(iii) TBAJ-587

**Figure 2:**
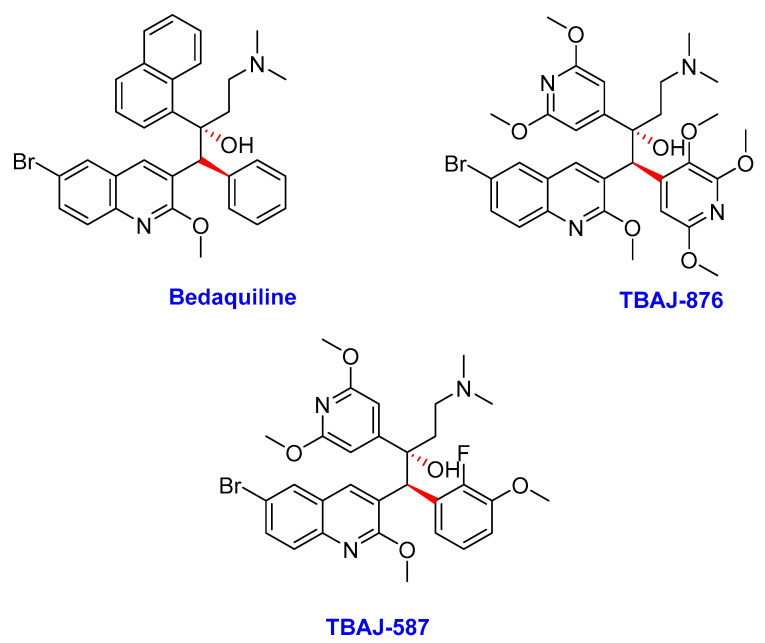
The chemical structures of diarylquinoline analogs in TB clinical studies.

TBAJ-587 is the second novel 3,5-dialkoxypyridine analog of BDQ discovered parallel with TBAJ-876 through medicinal chemistry campaigns aimed at improving the drug-likeness of BDQ. In contrast to TBAJ-876, the naphthalene moiety of BDQ is replaced with a less hydrophilic moiety, as evident in the structure of the three diarylaquinoline compounds below (Figure 2). Compared to TBAJ-876, TBAJ-587 has a high cLogP value (5.80), a slightly less log10 CFU of 5.2, and the same Cl values as TBAJ-876 in human microsome [78]. TBAJ-587 exhibits a substantially low AUC of 1.72 µg·h/mL, which is lower than that of BDQ [78]. It has been reported that BDQ’s AUC/MIC is the driver of its efficacy against murine TB [78]. 

### 2.3. Nitroimidazoles 

**Figure 3:**
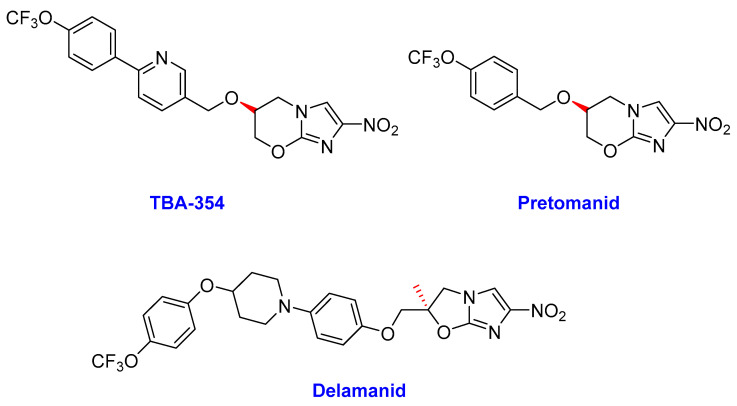
The chemical structure of nitroimidazoles undergoing clinical development as TB drugs.

Nitroimidazoles, related to metronidazole, are promising antimycobacterial agents. They are known to inhibit anaerobic bacterial activity and hence can be used to treat latent TB infection [80]. Currently, two nitroimidazole-based compounds, namely delamanid (DM) and pretomanid (PD) have been approved by FDA for TB treatment, while TBA-354 (Figure 3) is undergoing clinical trials for the treatment of TB as well.

(i) Delamanid

Delamanid (DM) is a new anti-Mtb drug derived from the nitroimidazole (nitro-dihydro-imidazooxazole) class of compounds [81]. Like BDQ, this drug was provisionally approved by the European Medicines Agency (EMA) in 2014 for the treatment of MDR-TB in adults [54]. As a prodrug, the pharmacological activity of DM is activated through bioreduction of its nitro group by the Mtb enzyme deazaflavin-dependent nitroreductase (Rv3547) to produce reactive nitrogen species [82]. These reactive intermediates, such as nitrogen oxides and desnitro-imidazooxazole derivatives, are associated with the inhibition of methoxy mycolic and keto mycolic acid biosynthesis [83]. This inhibition disrupts the Mtb cell wall and facilitates increased drug penetration [37], which therefore allow effective treatment within a shorter time frame [84].

Due to structural similarity with PD (another nitroimidazole derivative), there might be cross-resistance between these two drugs. The two compounds, fortunately, do not present cross-resistance with first-line TB drugs [57]. Although grouped under the D2 TB drug regimen category by the WHO, DM is currently undergoing Phase III clinical trials as part of a combination regimen for MDR-TB [22,85]. Phase III trials are being performed in seven countries, including the Philippines and South Africa, which are further considering phase I and phase II development for use in adolescents and children with TB [84].

DM exhibits potent, concentration-dependent in vitro activity against intracellular DS-Mtb and DR-Mtb strains with equal MICs in the range of 0.006–0.024 µg/mL [86]. The same study confirmed that the post-antibiotic effects of DM on intracellular Mtb after pulsed therapy were comparable with that of rifampicin [87]. Using a liquid culture system, Saliu et al. (2007) found that DM at 1 μg/mL showed intracellular killing activity similar to that of 3 μg/mL of rifampicin against a panel of drug-tolerant Ugandan Mtb isolates [86].

DM suffers from poor aqueous solubility; hence its oral bioavailability and Cmax are 2.7 times higher when administered with food compared to fasting conditions [83]. After oral administration, DM has a Tmax range of 4–5 h [87], while the steady-state concentrations are reached after 10–14 days [88]. DM and its metabolites are more than 99% protein-bound, with a large volume of distribution of around 1100 liters after 100 mg twice daily oral administration [82]. Metabolism occurs primarily by albumin in plasma, and the plasma half-life is 30–38 h in contrast to its metabolites which have a half-life ranging between 122–322 h [88]. Additional metabolism occurs via liver microsomal cytochrome (CYP) 450 3A enzyme. The primary metabolite, DM-6705, is formed by a reaction between the amino acid groups present in albumin and the C-5 of 6-nitro-2,3- dihydroimidazo[2,1-b]oxazole moiety of DM [83]. There are four major metabolites (M1–M4) that have been identified in the plasma of patients receiving DM [89], and the drug elimination is primarily through feces, with less than 5% of the drug excreted in the urine [82].

Generally, DM has a safe profile, but it causes mild-to-moderate QT prolongation. This side effect is attributed to its major metabolite (DM-6705), whose levels increase with dose [82]. Studies have shown that the DM effect on QT interval appears to be dose-dependent and that this effect is negligible at recommended therapeutic concentrations of DM (100 mg twice daily) [90]. In randomized clinical trials, where MDR-TB patients received DM 100 mg, 200 mg, or placebo each twice daily, the drug exhibited asymptomatic QT segment prolongation in both treatment groups (13.1% and 9.9%) compared to the placebo group (3.8%) [91,92]. Noteworthy, DM is not a substrate, inhibitor, nor an inducer of CYP450 isozymes; it is also not conjugated, acetylated, or excreted renally. Therefore, drug-drug interactions, if coadministered with other drugs for MDR-TB and/or HIV treatment, are not clinically significant. Regarding interactions with antiretrovirals, there is no significant data limiting coadministration of DM with first-line HIV therapy in regions of high MDR-TB/HIV coinfection. Coadministration of DM and BDQ generally showed good treatment outcomes, and the safety profile of this combination mirrored what has been observed in treatment with the individual drugs [92]. Further analysis showed that the risk of QT prolongation in DM treatment was strongly associated with hypoalbuminemia [54]; therefore, albumin under 2.8 g/dl is listed as a contraindication to DM treatment [82]. Additionally, few cases of hepatotoxicity amongst adult patients are reported; therefore, the EMA advises that treatment with DM should be avoided in patients with moderate-to-severe hepatic impairment [93]. Although there is limited toxicity data regarding DM treatment in pregnant women, it is not recommended for use in pregnant women or women of childbearing potential in the absence of a reliable form of contraception [83].

(ii) Pretomanid (PD)

Pretomanid (PD) is another drug belonging to the nitroimidazole class of compounds with a very complex mechanism of action. It is active against both replicating and non-replicating Mtb as well as DR-Mtb and DS-Mtb [94]. Like DM, PD requires intracellular activation by the deazaflavin-dependent nitroreductase to produce a des-nitro metabolite that generates reactive nitrogen species (including NO), which causes respiratory poisoning leading to a decrease in intracellular ATP and anaerobic killing [57]. In a similar action as isoniazid, PD kills aerobically by inhibiting cell wall mycolic acid biosynthesis [95]. Mutations in Mtb genes leading to either impaired prodrug activation (fgd1 and ddn) or the tangential F420 biosynthetic pathway (fbiA, fbiB, and fbiC) have been identified to cause resistance in PD [96]. Due to the contribution of such genes to the PD drug metabolism, these mutations are associated with the observed cross-resistance to 5-nitrothiophene (a tuberculostatic compound) as well as DM [96,97].

On 14 August 2019, PD received its first approval in the USA under the limited population pathway for antibacterial and antifungal drugs (LPAD) as part of a combination regimen with BDQ and linezolid (LZD). The drug was granted marketing authorization by the European Commission (EC) for treating XDR-TB, following completion of long-term follow-up of patients in the Nix-TB trial [98]. Thus, PD was listed as a prequalified medicinal product by the WHO [99]. Regulatory review in the EU is still pending due to incomplete evaluation of toxicity and efficacy of the BPaL regimen in the ZeNix trial, which enrolled participants with XDR-TB at sites in Africa and Eastern Europe [99,100,101]. Prior to this, PD continued to yield promising results from Phase II and III clinical trials conducted by the TB Alliance for various combination regimens [22].

PD has demonstrated activity against Mtb in vitro (including resistant strains and under anaerobic conditions) as well as in animal models of TB (as monotherapy or combination regimen) [102]. Furthermore, following 14-day dosing in DS-TB patients, PD (at dose levels of 200, 600, 1000, and 1200 mg/day) demonstrated bactericidal activity similar to that demonstrated by the standard first-line drug combination [22]. According to recent studies, the MIC of PD is in the range of 0.015–0.531 μg/mL against MDR and DS clinical isolates of Mtb [96], while its MIC_90_ against MDR- and XDR-TB is 0.063 μg/mL [103]. Moreover, in the continuation phase of a murine TB model, Jia et al. (2006) documented that PD maintained potent MIC values of 0.15–0.3 µg/mL and that these data are comparable to the activity of a combination regimen with isoniazid and rifampicin [104]. More intriguing results came from studies investigating PD as part of a novel TB regimen called PaMZ, comprising of PD, MFX, and pyrazinamide [105]. A comparative phase II trial study among patients with both MDR-TB and DS-TB, given PaMZ at 200 mg, showed superior bactericidal activity over standard therapy [106]. Another study also confirmed that PD is well tolerated at doses of 100–200 mg daily [107].

Furthermore, simulations of the PaMZ incorporating inter-subject variability were used to illustrate the effects of various covariate combinations (fasting/fed, HIV+, MDR-TB, bodyweight taking efavirenz) on some steady-state exposure criteria (including Cmax24h; and half-life). The Cmax24h yields were 0.22 µg/mL and 5.2 µg/mL for the fasting and fed subjects, respectively, with a half-life range of 10–30 h [108]. When either BDQ and/or LZD were added to the PaMZ, the curves were essentially the same as that of the reference value; this is an indication that BDQ and LZD together had little impact on PD exposure [108]. On the contrary, another comparative study involving PD alone and in combination with either efavirenz, rifampicin or lopinavir, showed a substantial reduction in plasma PD exposure by efavirenz and rifampicin but modest changes with lopinavir [109]. Moreover, in a murine model of TB, treatment employing triple combination of PD, BDQ, and LZD reduced bacterial counts in the lungs to a greater extent and resulted in fewer relapses at 2 and 3 months post-therapy compared to double combinations of the same drug cohort [110].

Notably, PD has been well-tolerated in humans at doses of 100–200 mg daily [107], with the Cmax reached about 4–5 h following oral administration taken with food [109,111]. After a single 200 mg dose of PD was administered with food to healthy adults, the mean Cmax of 2.0 μg/mL was achieved in a median 5 h, and the mean AUC∞ was 53 μg h/mL [100].

As with BDQ and DM, PD has been associated with QT interval prolongation [105]. A number of other adverse events have been reported in phase III trials which include peripheral neuropathy and anemia [112]. Nevertheless, PD remains a promising new agent with the potential to curb DR-TB, particularly in novel treatment regimens [106].

PD is mainly excreted in urine (53%), where ~1% of a dose is excreted unchanged, and in feces (38%), wherein the estimated mean apparent oral Cl after a single 200 mg dose under fed conditions in healthy adults is 3.9 h, and the estimated mean half-life is 17.4 h, which is in agreement with the PaMZ study findings [100].

(iii) TBA-354

The third nitroimidazo-oxazine derivative, which is bactericidal in nature and shows in vitro activity against both replicating and nonreplicating Mtb, is TBA-354 (TBA). It was subjected to Phase I clinical trials [113], parallel with a phase II randomized open-label trial by the TB Alliance [37].

Both in vitro and in vivo studies suggest that TBA has greater metabolic stability and thus shows a potentially better pharmacokinetic profile compared with other nitroimidazoles [113]. In vivo evaluation demonstrated a superior activity profile for TBA over PD when given at 100 mg/kg of body weight/day in acute and chronic murine infection models of TB [114]. The MIC of TBA was determined in another study that evaluated the antimycobacterial activities of PM analogs, where it was the most promising compound, having activity against both replicating and latent Mtb with MIC values of 0.006 μM and 0.27 μM, respectively [115]. Furthermore, Upton et al. (2015) discovered that following oral administration of a single dose of TBA at 3, 30, or 100 mg/kg, the Tmax was 2–6 h; the terminal half-life was relatively long 8–12 h, whereas the Cmax and AUC were dose-dependent, and their values ranged from 1.6–12.8 g/mL and 22.7–242 µg·h/mL, respectively [114]. However, as of January 2016, the only study of TBA had suspended recruitment with subsequent discontinuation of its development in March 2016 due to observations of side effects, including mild neurotoxicity, which was repetitive in all affected study subjects [116,117,118].

### 2.4. Oxazolidinones

**Figure 4:**
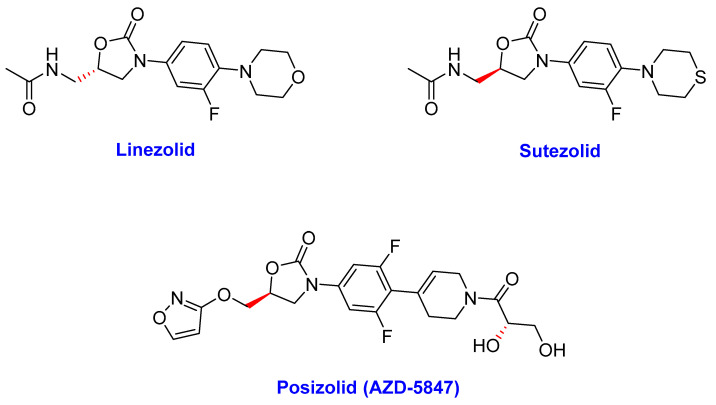
The chemical structures of oxazolidinones currently in TB clinical trials.

The oxazolidinones are a promising new class of synthetic antimicrobial agents with a unique mechanism of action, which includes inhibition of protein synthesis through binding at the P site of the ribosomal 50S subunit [119]. Binding to the 50S subunit distorts the site for formyl-methionyl tRNA; this inhibits ternary initiation complex formation and thus prevents initiation of translation [120]. Oxazolidinones display bacteriostatic activity against many important human pathogens, including DR-Mtb. In order to improve on TB regimens, the pharmaceutical researchers identified three drugs (LZD and its new chemical relatives, sutezolid, and AZD-5847) (Figure 4) to be of great potential [22]. Tedizolid (TZD), another oxazolidinone currently approved by the United States FDA for the treatment of acute bacterial skin and skin structure infections (ABSSSIs), is being evaluated for antimycobacterial activity [121,122,123]. Differences between the oxazolidinone drugs may be expected in their efficacies, pharmacokinetics, and adverse events that may occur during administration [37].

(i) Linezolid (LZD)

LZD is the first drug in the oxazolidinone class to be registered for the treatment of drug-resistant, Gram-positive bacterial infections mainly caused by Methicillin-Resistant *Staphylococcus aureus* (MRSA) and vancomycin-resistant *Enterococcus* species [124]. Recently, this drug is repurposed off-label to treat difficult cases of DR-TB as a recommendation in the WHO guidelines for the programmatic management of MDR-TB [37]. The WHO categorizes LZD under group C treatment for MDR-TB.

Randomized, double-blind, placebo-controlled phase I trials indicated that LZD is rapidly and completely absorbed after oral administration, reaching Cmax in 1–2 h after administration in fed healthy volunteers [125]. Another randomized controlled trial of culture-positive pulmonary XDR-TB cases showed a significantly higher probability of sputum culture conversions with LZD [126]. However, when LZD is given with high-fat food, Cmax is decreased by approximately 17% [127]. Cmax and AUC are dose-dependent, irrespective of the route of administration [125]. LZD inhibits the growth of MDR-Mtb isolates with MIC_90_ values of 0.3–1.25 µg/mL [22,124]. In a study on adults with drug-sensitive pulmonary TB, LZD (300, 900, and 1200 mg) exhibited dose-dependent bactericidal activity in all dose ranges across the populations studied. The best activity was observed with the highest once-daily dose of 1200 mg with a Cmax of 20.01 µg/mL, Tmax of 2 h, AUC_0–24_ of 228.4 µg·h/mL, a half-life of 5.4 h, and a MIC of 0.125–1.0 µg/mL [128].

Mutations at the G2061T and G2576T in the 23S ribosomal ribonucleic acid (rRNA) of Mtb can cause high levels of resistance to LZD, with resultant MICs of 32 μg/mL and 16 μg/mL, respectively. Isolates without mutation in 23S rRNA were found to be susceptible to LZD (MICs of 4–8 μg/mL) [129]. An important limitation of LZD is the frequent occurrence of side effects, especially myelosuppression and peripheral and/or optic neuropathy; however, this could be reduced by reducing the dosage from 1200 mg to 600 mg daily while maintaining efficacy [130]. Notably, peripheral neuropathy may be caused by long-term use and high-dose isoniazid, ethionamide, and cycloserine/terizidone; therefore, close monitoring of patients on these drugs is very important [54]. Dose-ranging studies have identified optimal LZD doses for use in combination therapy. This dose maximizes bactericidal activity while avoiding toxic effects [67]. Thus, LZD is being evaluated as a component of multiple novel TB regimens, including treatment shortening regimens. Its toxicity profile restricted its use beyond difficult-to-treat MDR-TB and XDR-TB cases in children; however, it is used for DS-TB treatment under special conditions [54].

(ii) Sutezolid

Sutezolid (SZD) is the second oxazolidinone, which is an analog of LZD, with preliminary evidence of superior efficacy against Mtb when compared to LZD both in vitro and in a mouse model of TB [131]. The drug is largely metabolized by flavin monooxygenases to sulfoxide and sulfone derivatives. The sulfoxide is the main metabolite and might contribute significantly to the parent drug’s activity [132]. SZD has the same mode of action as LZD, and it is anticipated that they vary in their pharmacokinetics and adverse effects [37]. The bactericidal activity against intracellular mycobacteria is mainly due to the parent SZD, whereas the sulfoxide metabolite (PNU-101603) contributes significantly to activity against extracellular mycobacteria [133]. In March 2017, the TB Alliance and the medicines patent pool (MPP) granted sublicensing of SZD for further development and commercialization in the treatment of both DS and DR-TB [134]. The drug is currently in phase II clinical development for the treatment of adults with both DR- and DS-pulmonary TB [135].

In vivo studies in the chronic mouse model of TB demonstrated that the addition of SZD to the standard TB regimen has the potential to significantly shorten the treatment. SZD did not only reduce the numbers of bacteria in the lungs more quickly but also led to a relapse-free cure with a shorter treatment duration [136]. Furthermore, in a sub-study with the murine model, the steady-state AUC for SZD and its metabolites observed with the 100 mg/kg daily dose was approximately 3-fold lower than that observed with LZD at 130 mg/kg [129]. This suggests that SZD monotherapy is 15 times more potent in vivo than LZD.

Combining SZD with existing first- and second-line anti-TB drugs results in dramatic increases in bactericidal activity, suggesting that it may have the potential to shorten the duration of chemotherapy for DS-TB as well as MDR-TB. For example, the addition of SZD to the first-line regimen of rifampicin-isoniazid-pyrazinamide increased the bactericidal activity, resulting in 2-fold lower colony forming units (CFU/mL), which is more than a simple additive effect [129]. In a clinical study where SZD was administered at doses of 600 mg twice daily or 1200 mg once daily for 14 days to patients with newly diagnosed DS-TB made promising findings. SZD showed effective sputum bactericidal activity regardless of dose. Interestingly, SZD treatment resulted in readily detectable whole blood bactericidal activity whether administered as a single or divided dose, and both doses produced maximal killing at 2–3 h post-dose administration [129]. The median MIC values for the parent drug and metabolite at baseline were ≤0.062 µg/mL and 0.500 µg/mL, respectively. These results corroborate in vivo simulation model study findings reported by Zhu and co-workers [137]. Comparison of the AUC_0–24_ values between the dosing regimens for both SZD and metabolite during a phase 1 study observed a mean AUC_0–24_ for SZD and metabolite in the range of 63–71% and 86–91%, respectively [136]. In contrast, the Cmax of SZD in single daily dosing increases proportionally with the dose (986 vs. 1972 ng/mL for 600 and 1200 mg, respectively), while the Cmax of the major metabolite increases gradually with doubling doses [131]. Assessment of blood and sputum data determined that extracellular killing is mainly due to the metabolite, whereas SZD act on intracellular mycobacteria, such as in the whole blood model [137]. Since the plasma half-lives of both the parent drug and metabolite are approximately 4 h, twice-daily split dosing would ordinarily be appropriate [133].

Orally administered SZD is rapidly oxidized in vivo to its metabolites which then undergo renal excretion [137]. SZD was safe and well-tolerated in phase I and II clinical studies and demonstrated potential efficacy in the treatment of TB. However, hemoptysis and peripheral neuropathy were observed in subjects post 600 mg dose treatment [131]. Phase I studies in healthy volunteers (600 mg twice daily for 28 days) revealed neither abnormal hematologic nor instances of ophthalmic neuropathy [129]. Therefore, recent adverse effects could be attributed to the standard TB treatment that followed after the trial in the case of peripheral neuropathy. Interestingly, SZD, unlike BDQ, MFX, DM, and PD described above, does not have effects on QTc interval irrespective of dose.

(iii) Posizolid (AZD-5847)

AZD-5847 discovered by Astra Zeneca, was originally intended as a broad-spectrum antibiotic but has now been repurposed as an anti-TB agent owing to its promising in vitro activity against DS-TB, MDR, and XDR strains of Mtb [22]. Having completed phase IIa trials and is likely to be advanced to the next phases, AZD-5847 is likely to be the third oxazolidinone repurposed as an anti-TB agent [138]. Despite showing the same mechanism of action as SZD and LZD, AZD-5847 demonstrated superior in vitro bactericidal activity against Mtb compared to that of LZD and has retained activity against MDR and XDR strains [105]. Moreover, AZD-5847 has additive activity when used in combination with other anti-TB drugs [139]. Consequently, this additive efficacy property against sensitive and resistant strains makes AZD-5847 a good candidate for trials of new multidrug therapies with existing or novel drugs [140].

Pre-clinical mouse studies determined that AZD-5847 obtained a dose-dependent kill rate constant, which was higher than that obtained for LZD [141]. Additionally, Furin et al. (2016) performed an EBA trial in adults with newly diagnosed sputum smear-positive pulmonary TB. Their findings using AZD-5847 (500 mg twice daily) and HRZE (combination of isoniazid, rifampin, ethambutol, and pyrazinamide) were 0.039 log_10_ and 0.163 log_10_ CFU/mL/day, respectively, by day 14 [138]. This is a significant decline in the number of CFU over time in favor of AZD-5847 at twice-daily doses of 500 mg, whereas there is no bacterial activity detected at 500 mg once daily doses of AZD-5847 [141].

Furthermore, Furin et al. (2016) identified a Tmax of 2–4 h. The Cmax, the C12h, and the AUC_0–12h_ were highest at a dose of 800 mg twice daily (11.54 µg/mL, 4.23 µg/mL, and 93.19 µg·h/mL, respectively) [138]. Moreover, AZD-5847 has a MIC of 1.0 µg/mL against both DS and DR strains of Mtb [138,141]. It is an orally bioavailable drug with increased bioavailability after food intake. It was 80% protein bound with a half-life of 7–11 h in healthy human subjects and is excreted mainly in feces [139].

AZD-5847 appears to be safe and well-tolerated in healthy volunteers, with nausea being the most common adverse effect occurring at higher dosages [57]. Moreover, few cases of hematologic, hepatic, gastrointestinal, and grade 1 QTcF prolongation were also observed [138]. Most of the adverse events associated with AZD-5847 are attributable to receiving higher doses of either 800 mg twice daily or 1200 mg daily [138].

(iv) Tedizolid (TZD)

TZD is a second-generation oxazolidinone that differs from other oxazolidinones by possessing a modified side chain at the C-5 position of the oxazolidinone nucleus, which confers activity against LZD-resistant pathogens [142,143]. In order to affect microbial activity, TZD is administered as the prodrug tedizolid phosphate, which is rapidly and extensively transformed to its active moiety by endogenous phosphatases in serum [144,145]. Previous studies show that TZD exhibits better antimicrobial potency, fewer adverse effects, good pharmacokinetics, aqueous solubility, and oral bioavailability compared to LZD [121,146]. All these enhanced activities are due to its optimized ring systems [143], as well as improved SAR and configuration enabling additional binding interactions relative to LZD and other oxazolidinone drugs [147]. Like other oxazolidinones, TZD acts through inhibition of bacterial protein synthesis by binding to the 23S rRNA of the 50S subunit of the ribosome [142,143], which prevents the formation of the 70S ribosomal initiation complex [121].

In vitro susceptibility studies of Mtb clinical isolates to TZD demonstrated potent activity with MIC value in the range of 0.0312–0.5 µg/mL compared to 0.25–4 µg/mL for LZD against drug-susceptible and resistant isolates [148]. This MIC range partly overlaps with in vitro data from another study wherein TZD exhibited MIC values against drug-susceptible, drug-resistant, and MDR in the range of 0.125–0.5 µg/mL, 0.06–0.5 µg/mL, and 0.125–0.5 µg/mL, respectively [149]. Deshpande et al. (2018) investigated responses to mono and combination TZD therapy in children with intracellular Mtb. They obtained the same MIC of 0.25 µg/mL from various Mtb strains using TZD as a monotherapy where it outperformed intracellular killing by LZD [150]. In combination with MXF, TZD demonstrated a bacterial burden elimination rate constant of 0.27 ± 0.05 per day and a half-life of 2.55 days [150], which corresponds with results obtained when MXF was used in combination with LZD from a previous study [151]. Additionally, combination treatment of TZD and CFZ is also known to have the potential to improve treatment efficacy and diminish the development of drug resistance [152].

Toxicity of oxazolidinones such as LZD is AUC driven, with a general mitochondrial inhibition at 94 mg.h/L; however, TZD is not associated with a downregulation of mitochondrial enzyme genes at AUC of 90 mg.h/L [150]. In order to fill the gap in Korean research, Kim et al. (2017) evaluated the PK, toxicity, and tolerability properties of TZD (200, 400, and 600 mg) single-dose oral and intravenous administrations in healthy Korean male subjects. The group observed the following mainly dose-dependent PK parameters which were similar in the binary routes: Tmax: 1.1–1.5 h; Cmax: 2679–3079 µg/L; AUC: 30,016–31,512 µg.h.L; half-life: 10.9–11.6 h; Cl: 5.5–5.7 [153].

Using the notion that in adult-type TB, 80% of bacteria are extracellular [154], Srivastava et al. (2018) studied the efficacy of tedizolid against extracellular semi-dormant bacteria in order to identify the sterilizing effect of TZD for Mtb treatment. The team utilized two different methods. According to the hollow fiber system model of TB (HFS-TB), microbial kill fell below limits of detection by day 42 using a once-weekly dose AUC of 424 mg.h/L, indicating a complete eradication of Mtb in the HFS-TB replicates [155]. Although the same AUC of 424 mg.h/L had the highest time to positivity (TTP), and the assay followed the same pattern as observed in the HFS-TB model, the TTP method demonstrated that extinction of the bacterial population was not achieved in 42 days [155]. However, intermittent administration of TZD achieved the best sterilizing effect as monotherapy, hence recommended for TB treatment programs.

### 2.5. Ethylenediamines

(i) SQ-109

**Figure 5:**
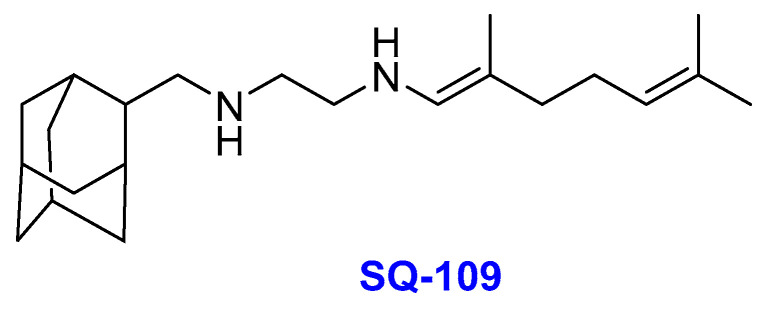
The chemical structure of SQ-109.

SQ-109 is a novel 1,2-ethylenediamine compound (Figure 5), an analog of ethambutol that is active against both DS-TB and DR-TB by targeting Mycobacterial membrane proteins Large 3 (MmpL3) in Mtb, with ultimate inhibition of cell wall formation [156,157]. MmpL3 transports trehalose monomycolates (TMMs) across cell envelop for subsequent incorporation into trehalose dimycolates or arabinogalactan during Mtb cell wall biosynthesis [158]. Although structurally similar to ethambutol, SQ-109 exhibits a different mechanism of action and has activity against ethambutol-resistant Mtb isolates [57,115]. The compound has gone through a phase II clinical trial [159].

In vitro studies on interactions of SQ-109 with other drugs showed that it does not interact positively with either ethambutol or pyrazinamide; however, SQ-109 was synergistic with isoniazid when both drugs were used at 0.5 MIC [140]. Additionally, SQ-109 at 0.5 MIC or 300 mg showed synergy with rifampicin [140,159]. Furthermore, in vivo tests of SQ-109 showed that this compound is able to cure induced TB in mice at a concentration 100 times lower than that of ethambutol, and there was a tolerable level of toxicity and good pharmacokinetics [160]. For example, the substitution of ethambutol (100 mg/kg) with SQ-109 (10 mg/kg) in the treatment of a mouse model of chronic TB improved the efficacy of a regimen containing rifampicin and isoniazid, with or without pyrazinamide regardless of treatment duration, resulted in 1.5 log_10_ lower CFU/mL/day and 25–30% decrease in time to the standard of care effects [161]. Regrettably, in a multi-arm, multi-stage trial testing high-dose rifampicin, all SQ-109-containing arms were terminated due to lack of sufficient efficacy [162].

Protopopova et al. (2005) determined SQ-109 MIC values in the range of 0.63–1.56 µg/mL against DS-Mtb, 0.9 µg/mL on ethambutol-resistant Mtb, 1.4 µg/mL on isoniazid-resistant Mtb and 0.7 µg/mL on rifampicin-resistant strain [160]. These data were further confirmed by da Silva et al. (2017), who demonstrated that SQ-109 exhibits MICs values of 0.7–1.56 µg/mL against both drug-susceptible and drug-resistant strains of Mtb [115].

It was observed that 30–40% of SQ-109 partially degrades in dog and human plasma, but it was stable in rat and mouse plasma, and the drug demonstrated oral bioavailability of 2.4–5% [163]. Elimination of SQ-109 is mainly through urine and slightly in feces, as observed in rats. In vitro microsomal stability analysis showed that the compound is metabolized through oxidation, epoxidation, and N-dealkylation [104]. Up to 58% of the drug was metabolized within 10 min of incubation with microsomes [163]. In mice studies, SQ-109 had been shown to be metabolized by the cytochrome P450 isoenzymes CYP2D6 and CYP2C19 [164]. Rifampicin induces the expression of CYP2C enzymes in humans; thus, it might lower the effective dose of SQ-109 [165].

SQ-109 appears to be safe and well-tolerated in human studies, with mild to moderate, dose-dependent gastrointestinal discomfort being the most frequently observed adverse events [159].

### 2.6. Imidazopyridine Amides

(i) Telacebec (Q203)

Q203 is a novel imidazopyridine discovered through phenotypic high-throughput screening performed against infected human macrophages and subsequent SAR optimization [61]. It has undergone parallel studies in phase Ib (evaluating safety, tolerability, and pharmacokinetics in healthy adults) and phase II (EBA treatment-naïve, sputum smear-positive patients with DS pulmonary TB) studies [166]. Q203 is also reported to be one of the new compounds under development for the treatment of DR-TB [167]. The compound exerts novel anti-mycobacterial activity by targeting the QcrB subunit of the cytochrome bc1 complex, thus interfering with the proper functioning of Mtb’s electron transport chain (ETC). This leads to inhibition of oxidative phosphorylation and ultimate death of Mtb [168]. Q203 is active against intracellular and MDR-Mtb [115]. According to Hoagland et al. (2016), the bc1 complex is an essential component of ETC, which drives ATP synthesis under aerobic conditions [72]. Therefore, Q203 causes a rapid depletion of intracellular ATP at an IC_50_ of 1.1 nM and interrupts ATP homeostasis in aerobic conditions [61,169]. These activities were all better than those observed with BDQ; hence, they justify the admirable killing profile of Q203 in a chronic mouse model of Mtb infection [72]. SAR for lead optimization of a series of imidazo[1,2-a]pyridine amide analogs, including Q203, revealed that the 3-carboxamide functionality, particularly the carboxamide linker with the *N*-benzylic group, is critical for mycobacterial activity [114].

Q203 possesses desirable pharmacokinetic and safety-profile data that allow for once-daily oral dosing, with 90% oral bioavailability in mice and an elimination half-life of 23.4 h [61]. In vivo efficacy assessment in a mouse model of established TB infection indicated that 10 mg/kg body weight of the drug, administered for 4-weeks, reduces 99.9% Mtb bacterial load [61]. These results were confirmed in an acute mouse model, showing comparable effects of Q203 to that of BDQ and isoniazid [170]. Additionally, in vivo mice studies evaluated pharmacokinetic properties of Q203 after an oral dose of 10 mg/kg and discovered Cmax and AUC of 1490 ng/mL (reached in 2 h) and 44,100 ng.h/mL, respectively [167]. Q203 has shown high potency against DS-Mtb H37Rv strain with MIC value of 0.0027 µg/mL as well as against MDR and XDR Mtb clinical isolates with MIC_90_ < 0.00043 µg/mL [61].

In order to ascertain its potential for synergy during combination therapy with other drugs, Q203 was evaluated for cytochrome P450 inhibition with five different isoenzymes and showed no inhibition to any of the isoenzymes tested [126]. This indicates that the drug has a low risk of drug-drug interactions. Finally, Q203 showed no record for QT interval prolongation [61].

(ii) TB47

**Figure 6:**
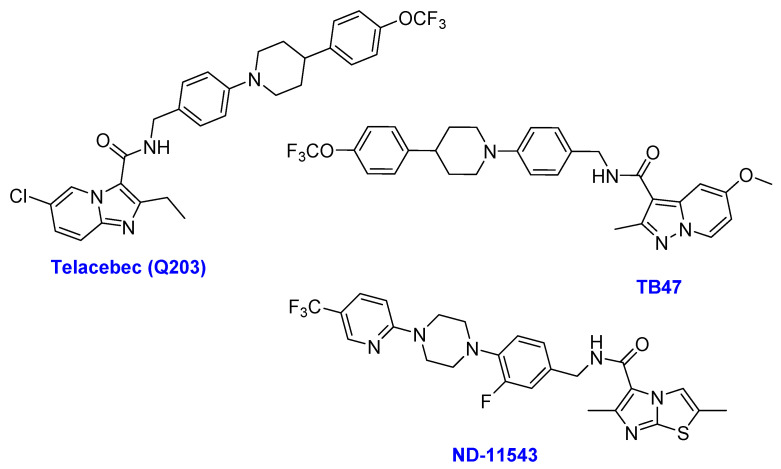
The structures of Q203 and its analogs (TB47 and ND-11543) that show potential as novel TB agents.

The discovery of Q203, which portrays good activity against Mtb, has inspired the search for several related analogs (Figure 6). This eventually led to the discovery of TB47, a pyrazolo[1,5-a]pyridine analog of Q203 discovered through scaffold hopping [171]. Like Q203, TB47 acts by targeting the QcrB subunit; therefore, the pyrazolopyridine carboxyamides class is identified as a novel scaffold for QcrB inhibitors [172]. TB47 blocks the cytochrome bc1 complex, thereby causing a reduction in intracellular ATP and eventually inhibiting the growth of Mtb [172,173]. In order to understand its mode of action, TB47 was docked into the quinol oxidation site (Qp) of the QcrB, whereby potential hydrogen bonding interactions between the amide of TB47 and the glutamate residue (Glu314) of the Qp binding site were observed [174]. 

TB47 was found to exhibit strong potencies against the drug-sensitive H37Rv strain of Mtb with antitubercular effects comparable to those of rifampicin [171], which encouraged further studies to validate the drug’s therapeutic potential. Upon evaluation against several clinical isolates, TB47 displayed growth inhibition with MIC values between 0.016 and 0.500 µg/mL. Thus, the drug shows promising treatment potential for either DS-, MDR- and/or XDR-Mtb H37Rv strains [172].

Furthermore, acute toxicity studies done in both human cell lines and rats did not demonstrate any measurable toxicity [172]. Owing to the good safety profile as well as its bacteriostatic effect during monotherapy, Lu et al. (2018) further explored synergistic activity between TB47 and other drugs. They observed that TB47 exhibited potent synergy with sub-therapeutic doses of PZA and rifampicin, resulting in a 4- and 5-fold reduction in lung CFU compared with PZA and rifampicin monotherapy, respectively [172]. This was supported in both in vitro and macrophages, whereby TB47 showed a strong synergistic bactericidal activity with CFZ against Mtb [173]. Moreover, TB47 was found to contribute significantly to both bactericidal and sterilizing activities of the second-line regimen ALEZC (amikacin (A), LVF (L), ethambutol (E), and PZA (Z), CFZ (C), leading to the discovery of the new regimen ALEZCT (where TB47 = T) [173]. It has been proposed that new regimens containing TB47 and CFZ may shorten the duration of MDR-TB from 9 months to 5 months [173,175].

(iii) ND-11543

Further modifications of the core of the imidazo[1,2-a]pyridine led to the discovery of another chemical entity (ND-11543) exhibiting antitubercular activity. ND-11543, a member of the imidazo[2,1-b]thiazole-5-carboxamides class, was also designed along with TB47 by the scaffold hopping strategy based on the therapeutic potential demonstrated by Q203 [176]. The imidazo[2,1-b]thiazole-5-carboxamides also act by targeting oxygen-dependent respiration through the QcrB-bc1 complex with profound selectivity against replicating and drug-resistant Mtb strains [176].

ND-11543 exhibited a low MIC value of 0.01 µg/mL in vitro and 0.0625 µg/mL against Mtb in macrophages [174,176]. Additionally, the compound demonstrates low toxicity and a half-life of 28.4 min in human liver microsomes [177]. Treatment of a chronic TB mouse model with 200 mg/kg of ND-11543 without a CYP-inhibitor led to a slight reduction in CFU of 0.30 and 0.36 log_10_ in lung and spleen, respectively. The addition of the CYP-inhibitor improved the reduction in bacterial load, exhibiting 0.59 and a 0.77 log_10_ CFU reduction in the lung and spleen, respectively [177]. Furthermore, Moraski et al. (2020) also observed AUC_0–24_ of 37,250 ng.h/mL and a Cmax of 2411 ng/mL for ND-11543 (in the presence of a CYP-inhibitor) [177] compared to Q203 with AUC_0–24_ of 44,100 ng.h/mL at the same oral dose of 200 mg/kg in a chronic murine model [61].

### 2.7. DprE1 Inhibitors

**Figure 7:**
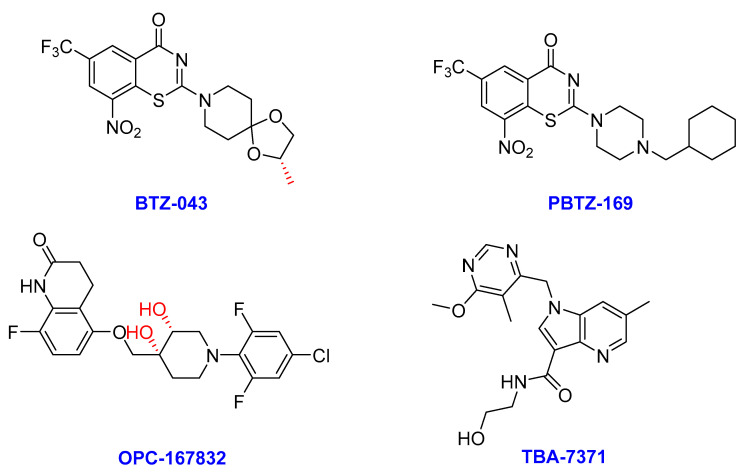
The chemical structures of novel DprE1 inhibitors under clinical development for Mtb treatment.

Decaprenyl-phosphoribose epimerase (DprE1) catalyzes the first step in the epimerization of decaprenyl-phosphoryl-β-D-ribose to decaprenyl-phosphoryl-β-D-arabinose, the important arabinose precursor for the synthesis of arabinogalactan and lipoarabinomannan, critical components of the mycobacterial cell wall [80]. Below is the update on pharmacokinetic and safety parameters of new DprE1 inhibitors (Figure 7) that are investigated at advanced levels against various Mtb strains.

(i) BTZ-043

BTZ-043 is a piperidine-containing benzothiazinone prodrug activated through bioreduction of its nitro group by mycobacterial enzymes (including DprE1) to yield a nitroso metabolite [115,165]. This metabolite reacts with the thiol group of a cysteine residue (Cys387) in the substrate-binding site of DprE1 to form a covalent bond that irreversibly inhibits DprE1, thus classifying BTZ-043 as a suicide inhibitor [178].

In vitro combination studies demonstrated high selectivity of BTZ-043 against Mtb, good activity against DS, MDR, and XDR-Mtb strains, as well as a good toxicity and safety profile [179,180]. The ongoing phase Ib/IIa clinical trials are evaluating the safety, tolerability, EBA, and pharmacokinetics of BTZ-043 in patients with DS-Mtb [179]. Further in vitro studies indicated that BTZ-043 acts additively in combination with rifampicin, isoniazid, PD, and MFX and is synergistic in combination with BDQ [140]. The MIC of BTZ-043 against replicating and non-replicating Mtb are 1 ng/mL and <10 ng/mL, respectively [115,178]. According to da Silva et al. (2017), BTZ-043 possesses significantly lowers MICs than the MICs of all currently used TB drugs and drug candidates [115]. BTZ-043 is less effective under both auxotrophy and starvation model systems, implying that it blocks a step in the active metabolism, similar to isoniazid [181]. Mutation at the Cys387 position of DprE1 is the main mechanism of resistance to BTZ-043 [72]. Results from toxicological studies on uninfected mice indicated that even at the highest dose tested, BTZ-043 showed no adverse effects after one month [181].

Investigation of the pharmacokinetics of BTZ-043 using 5 mg/kg oral administration on rats resulted in rapid absorption: Tmax: 0.25 h; half-life: 1.22 h; Cmax: 543 ng/mL; AUC_0–__∞_: 899 ng.h/mL [182]. BTZ-043 possesses an MIC value of 1 ng/mL against replicating Mtb [181]; a value superior to those of existing TB drugs such as isoniazid (0.02–0.2 mg/mL) and ethambutol (1 to 5 mg/mL) [183].

(ii) Macozinone (PBTZ-169)

The extensive SAR studies on BTZ-043 led to second-generation benzothiazinones namely 2-piperazinyl-benzothiazinones [184]. PBTZ-169 is a piperazinyl-benzothiazinone based structure that offers multiple advantages over its predecessor (BTZ-043), including a simpler synthetic route, improved pharmacodynamics properties, and better potency against both DS-Mtb and DR-Mtb [80,165]. Moreover, in vitro cytotoxicity assessments done using HepG2 human cell line indicated that PBTZ-169 is 10-times less cytotoxic than BTZ-043 (i.e., median toxic dose (TD50) of 58 µg/mL and 5 µg/mL, respectively) [184]. PBTZ-169 exhibits the same mode of action as BTZ-043, which is the inhibition of DprE1 function by the formation of a covalent bond to the active site Cys387 residue. This can be confirmed by the presence of cross-resistance between BTZ-043 and PBTZ-169 for BTZ-resistant strains of Mtb, *M. bovis* BCG, and *M. smegmatis* [185]. However, the ease of chemical synthesis of PBTZ-169 is facilitated by the absence of a chiral center which is present in BTZ-043 [165]. Additionally, it is hypothesized that the presence of the flexible cyclohexyl substituent at N4 of piperazine on PTBZ-169 offers enhanced potency [80]; by making it more adaptable to the active site, hence its higher affinity and faster inactivation of DprE1 as compared to BTZ-043 in a chronic TB mouse model [184].

Due to many superior features of PBTZ-169, it was subjected to phase II EBA trials [67]. An in vitro study using Mtb clinical isolates discovered that PBTZ169 exhibits excellent in vitro activity against MDR-TB and XDR-TB with MICs less than 0.25 mg/L [186]. In a mouse model of chronic TB infection study, a combination of PBTZ-169 with pyrazinamide and BDQ proved to have better efficacy than the standard triple therapy of isoniazid, pyrazinamide, and rifampicin [184]. PBTZ-169 was also shown to have a synergistic effect with BDQ and CFZ [168]. Generally, PBTZ-169 has no activity on non-replicating conditions [187]; however, these synergistic effects are observed against both replicating and non-replicating Mtb H37Rv in vitro, as well as in a mouse model of chronic TB [187]. This characteristic is explained in two possible ways; weakening of the cell wall by DprE1 inhibition leading to better penetration of BDQ and easier access to its target, ATP synthase [184]. As such, PBTZ-169 is a promising candidate for novel TB treatment regimens.

In a mouse model of chronic Mtb infection, the bacterial lung burden was reduced by 2.0, 4.0, and 4.6 log_10_ units after 28 days of treatment with PBTZ-169, CFZ, and a combination of PBTZ-169/CFZ, respectively [187]. This was further corroborated in an experiment with Mtb chronically infected mice, where the combination of PBTZ-169, BDQ, and pyrazinamide was more effective in reducing the number of CFU in lung and spleen than the standard treatment; whereas, the effect was similar between isoniazid and PBTZ-169 after 4 weeks of treatment [184]. PBTZ-169 displays comparable bactericidal activity to isoniazid; in contrast to isoniazid, a significantly dose-dependent activity was observed in comparison to BTZ-043 in both the lungs and spleens of mice at 25 mg/kg [184].

Although PBTZ-169 was found to have poor solubility in water (0.9 g/L) [165], further studies demonstrated that the drug is more soluble in acidic environments. Hence, it is absorbed primarily in the stomach (at a pH range of 1–2) as observed in animals and healthy volunteers [185] and partly in the intestines at a more rapid rate than BTZ-043 [165]. Therefore, increased bioavailability and exposure of PBTZ-169 are enhanced by prolonging its presence in the stomach. During clinical trials, PBTZ-169 demonstrated substantially and statistically significant increased pharmacokinetic parameters when 640 mg dose was administered in fed conditions; AUC_0–8_ increased 3.45-fold and Cmax increased 2.29-fold [185]. The drug was absorbed and cleared from the body more slowly when administered in fed conditions compared to the fasting conditions; hence, the preferred route of PBTZ-169 intake is oral administration after meals [188]. In addition, the actual plasma concentration of PBTZ-169 could be affected by its ability to undergo biotransformation into several metabolites. Of interest, the hydroxyl- and oxo-metabolites of PBTZ169 have been found to display good anti-TB activities in vitro [189]. For example, H2-PBTZ169 was reported to be highly unstable, easily reverting back to PBTZ-169 upon air oxidation, which would affect the actual concentration of the parent drug in biological fluids [190].

(iii) OPC-167832

OPC-167832 is a compound under the carbostyril chemical class that is being developed by Otsuka Pharmaceutical Co, Japan [165]. Similar to BTZ-043, OPC-167832 exhibits bactericidal activity against both replicating and non-replicating Mtb bacilli [191]. However, in contrast to benzothiazinones (BTZ-043 and PBTZ169), the 3,4-dihydrocarbostyril derivative (OPC-167832) does not form a covalent adduct with DprE1 [192]. Because OPC-167832 and other non-covalent DprE1 inhibitors do not contain a nitro group, they inhibit DprE1 through electrostatic or hydrophobic interactions with different amino acids within the active site of DprE1 [165]. Noteworthy, SAR studies on BTZ-043, which is characterized by a nitro group, suggest that this chemical group is fundamental for effective anti-mycobacterial activity [193]. The difference in binding sites between these inhibitors was confirmed by analysis of the points of mutations in the amino acid chains. It was hypothesized that the distinct means of DprE1 binding (i.e., covalent vs. non-covalent binding) might affect the level of resistance [181]. Therefore, while a mutation at Cys387 on DprE1 leads to high-level BTZ-043 resistance, the mutation in the same position produces only moderate level resistance to OPC-167832 [194]. Additionally, the non-covalent inhibition may overcome potential toxicities and resistance associated with the covalent DprE1 inhibitors [192]. Furthermore, a whole genomic DNA sequencing revealed mutations of two genes, rv0678 and rv3790, in OPC-167832 resistant isolates [195]. These genes were reported as repressors for the MmpS5/Mmpl5 efflux pumps of Mtb and DprE1, respectively; therefore, mutations in them drive resistance to OPC-167832 [196].

OPC-167832 was documented to be undergoing phase I/II clinical study with multiple oral doses in patients infected with DS pulmonary Mtb [179]. OPC-167832 is a lipophilic, bactericidal compound active against DS, MDR, and XDR intracellular Mtb strains [165]. The compound is known to exhibit potent MIC in the range of 0.00024–0.002 µg/mL against Mtb H37Rv, which are lower than those of rifampicin (0.1–0.2 µg/mL) [179,197].

After oral administration in mice, OPC-167832’s plasma level reached a peak at 0.5–1.0 h with a half-life of 1.3–2.1 h. Its distribution in the lungs was approximately two times higher than that in plasma and showed dose-dependent Cmax and AUCt in both organs [195]. In addition, Hariguchi and co-workers observed a dose-dependent decrease in lung CFUs from a dose of 0.625–2.5 mg/kg in a mouse model of TB [195]. Moreover, combination studies discovered that a new drug regimen of OPC-167832 and DM displays good synergism than a standard TB-drug regimen in the mouse model [191]. This suggests a potential for treatment shortening opportunities in DS-TB and/or DR-TB patients. Furthermore, combinations of OPC-167832 with DM, BDQ, or levofloxacin exhibited significantly higher efficacies than that of the independent agents [195].

(iv) TBA-7371

Another prospective DprE1 inhibitor on Mtb treatment trial is TBA-7371, a derivative of 1,4-azaindole developed through scaffold morphing of an imidazopyridine compound with good MIC but low minimum bactericidal activity (MBC) against Mtb [198]. In order to optimize the bacterocidal activity of the imidazopyridine used in the scaffold morphing, Shirude and co-workers established a MIC-based SAR against Mtb. They identified three points of diversification for the resulting 1,4-azaindoles, these are: (i) amide side chain which is considered essential for various intermolecular hydrogen-bonding interactions with the DprE1 and hence potency; (ii) a hydrophobic group and (iii) core ring hydrophilic substitutions, both contributing to cellular potency as well as physicochemical and in vitro safety properties [198]. During these early SAR studies, it was suggested that, unlike BTZ-043 and PBTZ-169, TBA-7371 is a non-covalent inhibitor of DprE1 [198,199]. In contrast, it was recently revealed through computational docking studies that the compound exhibits both non-covalent and covalent binding interactions to DprE1 [179], possibly acting in the same mode as PBTZ-169 described above.

Following successful completion of phase 1 clinical trial study, which was aimed at evaluating its safety, tolerability, pharmacokinetics, and drug-drug interactions, TBI-7371 has undergone phase II trial for further clinical studies relating to escalating doses [185]. TBA-7371 shows bactericidal effects with a MIC range of 0.78–3.12 µg/mL against DS and DR clinical isolates of Mtb [199]. The drug displays high permeability and does not inhibit the CYP450 isoenzymes [200]. Consequently, TBA-7371 shows oral exposure and bioavailability of 86% and 100% in rats and dogs, respectively. It displays AUCs in the range of 166–240 µg·h/mL, which predicts excellent bioavailability in humans [199]. Additionally, the study demonstrated that TBA-7371 does not exhibit toxicity in a human monocytic cell line (THP1) and the hERG channel up to a concentration of 100 and 33 µM, respectively. Combination studies with various first- and second-line anti-Mtb drugs and clinical/pre-clinical TB candidates in vitro showed no antagonism between drugs with good bacterial load reduction in the lungs CFU by ± 1.5 log_10_ [199,200]. Further studies suggest an overall low risk of cardiovascular toxicity as well as prove the potential for combination TB therapy [165].

Finally, despite showing excellent in vivo and in vitro efficacy against Mtb as well as no major safety liabilities with low Cl in mice, rats, dogs, and humans, the weak inhibition of host phosphodiesterase 6 (PDE6) observed with TBA-7371 may lead to impaired visual acuity [199]. Additionally, some resistance to TBA-7371 and other azaindoles was observed in strains overexpressing Mtb DprE1 or carrying the mutation Y314H in DprE1, but not in strains with a mutated Cys387 in DprE1 [198].

### 2.8. Caprazamycins

**Figure 8:**
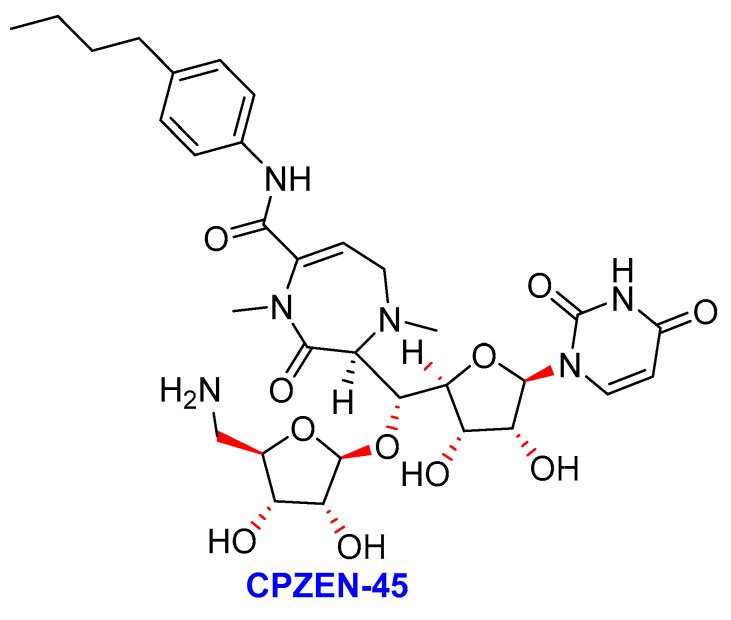
The chemical structure of CPZEN-45, a potential drug candidate for TB therapy.

Caprazamycins (CPZs) are members of a class of naturally occurring 6′-N-alkyl-5′-β-O-aminoribosyl-glycyluridine (liponucleoside) antibiotics isolated from *Streptomyces* spp. MK730-62F2 [201]. The liponucleoside mode of action involves targeting the phospho-MurNAc-pentapeptide translocase (MraY, translocase I), which catalyses the attack of UDP-N-acetylmuramoyl (UDP-MurNAc) pentapeptide by the undecaprenol monophosphate in the bacterial cell membrane providing the lipid I (undecaprenyl-pyrophosphoryl-MurNAc-pentapeptide) [202]. Their core skeleton is the (+)-caprazol composed of an *N*-alkylated α-5’-(ß-1-O-aminoribosyl)-uridinyl-glycine cyclized into a seven-membered diazepanone ring, an amino ribose, a uridine, and a fatty-acid side chain [203,204]. The CPZs have shown excellent anti-mycobacterial activity in vitro against DS- and MDR-Mtb strains, exhibiting MIC of 3.13 µg/mL without presenting significant toxicity in mice [201]. CPZEN-45 is a novel caprazamycin drug candidate that displays potential antibacterial activity against Mtb strains and thus attracted interest for clinical studies recently. Structurally, CPZEN-45 (Figure 8) and other caprazamycins are composed of a seven-membered ring containing two nitrogen atoms, amino ribose, and uridine moieties [205]. In contrast to the other caprazamycins, CPZEN-45 has an α,β-unsaturated amide instead of a fatty-acid side chain.

(i) Caprezene-4-butylanilide (CPZEN-45)

CPZEN-45 is a novel semi-synthetic derivative of naturally occurring caprazamycin produced by *Streptomyces* spp., which is currently under pre-clinical development [179]. The novel liponucleoside antibiotic was discovered through SAR studies aimed at improving poor oral bioavailability and low hydrophilicity generally associated with caprazamycins [72]. Notably, CPZEN-45 exhibits potent antibacterial activity against several DS, as well as MDR and XDR strains of Mtb in murine models and exhibits synergistic effects with other TB drugs [179,205]. Generally, caprazamycins are effective against a broad spectrum of bacteria; in contrast, CPZEN-45 has a narrow spectrum and particularly effective against slowly growing mycobacteria, implying a possibility of different modes of action between these inhibitors [206]. An intensive target identification study by Ishizaki and co-workers validated that CPZEN-45 is effective against WecA (Rv1302) of Mtb (an ortholog of TagO) that is involved in the biosynthesis of the mycolylarabinogalactan of the cell wall of Mtb [206]. TagO is a membrane protein, which mediates the first step in the biosynthesis of teichoic acid, the transfer of GlcNAc-1-phosphate from UDP-GlcNAc to undecaprenyl phosphate with the formation of undecaprenyl-P-P-GlcNAc [206].

CPZEN-45 was found to be active in vitro against both replicating and non-replicating Mtb with an additive effect against DS- and MDR-Mtb when administered with other anti-TB drugs [12]. The MIC values against replicating and non-replicating Mtb were observed in the range of 3.13–12.5 µg/mL and 6.25–12.5 µg/mL, respectively [207]. Takahashi et al. (2013) further concluded that mice intravenously infected with acute TB and treated with 200 mg/kg of CPZEN-45 had a 1–1.5 log_10_ CFU better reduction in the lungs than the mice receiving the lowest dose (6.3 mg/kg) after 30 days [207]. This suggests that the novel drug activity is dose-dependent. Moreover, the same group did a comparison study using a single dose of CPZEN-45, isoniazid, and/or rifampicin, as well as combination regimens isoniazid/rifampicin/CPZEN-45 and isoniazid/rifampicin in mice intravenously infected with the DS-Mtb H37Rv strain. The results indicated that the triple regimen exhibited at least a 1 log_10_ better killing of Mtb than the isoniazid/rifampicin combination, while the treatment with CPZEN-45 was as effective in reducing lung burden as isoniazid and better than rifampicin alone [165].

In addition, in order to enable inhalation administration in humans, a powdered co-product of capreomycin and CPZEN-45 has been developed using a spray drying technique [208]. Subsequently, a study confirmed that CPZEN-45 is efficiently absorbed by lung tissue through inhalation and can reach therapeutically relevant concentrations at the primary site of Mtb infection [209]. In summary, with no acute cytotoxic effects at concentrations up to 3 mg/mL [209], a good safety profile, exhibition of good solubility, and cell permeability, CPZEN-45 proves to be a promising antituberculosis drug candidate [206].

### 2.9. Riminophenazine

**Figure 9:**
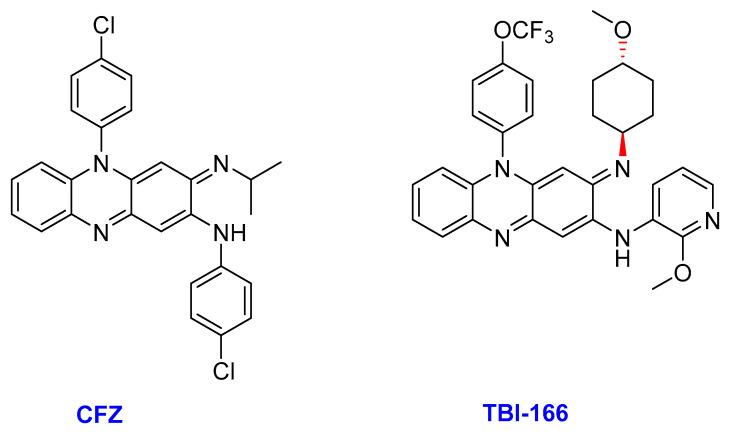
The chemical structures of riminophenazines: CFZ and TBI-166 with potential anti-TB properties.

Riminophenazines were discovered following structural modifications aimed at improving the activity of a group of compounds known as anilinoaposafranine, which were found to be highly effective against tubercle bacilli [210,211]. Riminophenazine is a class of tricyclic heterocycles composed of a central phenazine ring substituted at a nitrogen, whereas the “R” substituents are mainly at the imino moiety (NH), hence the designation “rimino” compounds (Figure 9) [212,213]. Among the analogs prepared was a compound (2-chloro-anilino-5-p-chlorophenyl-3,5-dihydro-3-isopropyl ininophenazine) initially called B663 and later changed to Lamprene or CFZ [212].

Although many studies confirmed that CFZ and its analogs display high in vitro antitubercular activity, further experiments were halted due to findings that the drug was not active in animal models like guinea pigs and monkeys [211,214]. However, subsequent studies gathered that the lack of activity of the drug in vivo was due to poor oral absorption in guinea pigs and monkeys [214]. Although this was not the case in hamsters and mice, having demonstrated high in vivo activity against TB following oral administration, it has not reversed the reputation the drug suffered due to earlier findings [215].

Several years later, based on the fact that CFZ is accumulative inside the cells of the mononuclear phagocyte, it was assumed that it should treat an intracellular disease such as leprosy [216]. Thus, the proposition that the mechanism of action of CFZ is through intracellular redox cycling where the drug is reduced by the ETC enzyme type 2 NADH dehydrogenase (NDH-2) with the generation of bactericidal reactive oxygen species such as hydrogen peroxide [213,217,218]. To this effect, CFZ has been used for the treatment of leprosy since 1969 and later repurposed as an antimycobacterial agent [219]. However, the application of CFZ in TB treatment is limited mainly by its side effect of skin discoloration as well as some of its physicochemical properties, including extreme high lipophilicity [212,220].

(ii) TBI-166

The repurposing of CFZ, a riminophenazine antibiotic, as an important component of the new short-course MDR- and DS-Mtb regimens [220] subsequently led to the development of TBI-166, a pre-clinical candidate for TB drug development identified from a series of CFZ analogs [46]. SAR studies indicated that TBI-166, the synthetic analog, demonstrates enhanced in vitro potency compared to CFZ against replicating Mtb, as well as panels of DS and DR clinical isolates of TB [220,221,222]. In addition, TBI-166 was reported to act against intracellular and nonreplicating bacilli [115]. TBI-166 was recently approved by the Chinese National Medical Products Administration (NMPA) to enter phase I clinical trials. Its mechanism of inhibition is via the non-proton-pumping type II NADH dehydrogenase (NDH-2) and the QcrB complex, as observed in Q203 [223]. Hence, it acts by blocking DNA synthesis [115].

Initial investigations indicated that TBI-166 was exclusively effective against DR clinical isolates of Mtb in vitro and showed greater than 1 log_10_ CFU/mL reduction than the CFZ at a dose of 20 mg/kg in Mtb H37Rv-infected mice for 3 weeks [222]. Recently, in an acute infection model, the mean lung CFU counts for various doses of TBI-166-treated groups were 2.2–4.2 log10 units lower than those for the untreated control and the rifampicin-treated groups [220]. The study further reported MICs of TBI-166 against replicating Mtb H37Rv, DS, and DR clinical isolates of Mtb to be 0.063, <0.005–0.15, and 0.01–0.2 µg/mL, respectively.TBI-166 exhibited dose-dependent bacteriostatic rather than bactericidal anti-TB activity at doses ranging from 10–80 mg/kg. Notably, the study observed that the bacteriostatic activity rendered by 20 mg/kg TBI-166 in mice, as well as its in vivo efficacy at the same dose, were equivalent to those of CFZ. It is noteworthy to mention that CFZ is bacteriostatic with a slow onset of bactericidal activity. The study also validated findings that TBI-166 significantly reduced skin discoloration compared to CFZ, as previously reported [221].

These improved physicochemical properties with reduced lipophilicity may be attributed to the replacement of the C-2 phenyl ring in CFZ with 2-methoxypyridyl group [220]. Furthermore, the plasma concentration of TBI-166 was observed to increase than that of CFZ, 3 h after dosing, suggesting that TBI-166 is eliminated faster and possesses a plasma half-life of about 45 h [223], which is shorter than that of CFZ of about 65 h; according to unpublished data in a single-dose pharmacokinetics study by Xu and co-workers.

TBI-166 is not associated with adverse events below a dose of 3000 mg/kg, and it lacks cross-resistance with currently used anti-TB agents, indicating its potential utility against MDR strains. However, CFZ/BDQ-resistant mutants harboring mutations in rv0678 were suspected to be partly resistant to TBI-166 [220]. In summary, the efficacy of TBI-166, along with its decreased potential for accumulation and tissue discoloration, signifies a promising candidate for the treatment of TB.

### 2.10. Pyrroles

**Figure 10:**
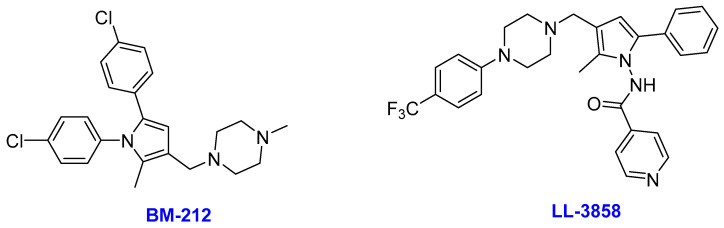
The chemical structures of pyrrole drug candidates being investigated for TB treatment.

A pyrrole is one of the most prominent heterocycles and are present in a broad range of natural products. For example, it is a structural component of heme and chlorophyll (pigments essential for life), chlorins, vitamin B12, and bile pigments (biliverdin and bilirubin) [224]. In addition, pyrrole derivatives have been found to possess a wide spectrum of activities like antibacterial, antifungal, antitubercular, anti-inflammatory, analgesic, anti-tumor, anti-epileptic, anti-viral, anti-hypertensive, and anti-diabetic agents [225,226]. This therapeutic diversity motivated new efforts in searching for novel pyrrole derivatives with improved biological activity and multiple applications in the pharmaceutical industry [226]. Pyrrole derivatives BM-212 and LL-3858 (Figure 10) are the two members of this class identified to exhibit promising antimycobacterial activity recently.

(i) BM212

The anti-tuberculous activity of the pyrrole class was first described in 1998 [227]. BM212, a 1,5-diaryl-2-methyl-3-(4-methylpiperazin-1-yl)methyl-pyrrole is the most potent pyrrole derivative studied so far; thus, it can be used as a lead for the preparation of new and more efficacious antimycobacterial drugs [63]. This compound exhibited MIC in the range of 0.7–1.5 µg/mL against several strains of Mtb, including the resistant strains [227].

The mode of action for pyrroles is not well understood, although efforts are underway to overcome this void. The trehalose monomycolate exporter, MmpL3 protein (Rv0206c), a member of the MmpL family, was identified as the cellular target after genetic analyses on spontaneous mutant resistance to BM212 were performed [228]. MmpL3 belongs to the resistance, nodulation, and cell division protein superfamily and is likely involved in trehalose monomycolate flipping across the inner membrane of Mtb [229]. This suggests that BM212 would block the transport of mycolic acids that are essential for the development of the mycobacteria cell wall, thereby inhibiting their growth [228].

Like other pyrroles, BM212 suffers from poor pharmacokinetic properties (Cl and microsomal stability) and significant cytotoxicity; hence it is still in the lead optimization studies for TB treatment. As such, the identification of novel pyrrole analogs with improved solubility and bactericidal activity against MDR and XDR mycobacteria continues [230].

(ii) LL-3858

The potent activity displayed by BM212 inspired the researchers at Lupin, India, to synthesize a series of pyrrole derivatives that led to the discovery of LL-3858 [231]. LL-3858 is a substituted pyrrole derivative that has completed phase I clinical studies for the treatment of TB and undergone phase II clinical trials [80,232]. The therapeutic target of LL-3858 against Mtb is not yet known; however, its structure includes an isonicotinyl hydrazine moiety, yet the compound exhibit activity against isoniazid-resistant strains, suggesting that it might possess different mechanisms of action from that of isoniazid [80]. Moreover, SAR of LL-3858 analogs revealed that the isonicotinyl hydrazine ring is not crucial for activity, though the phenyl ring at N-4 piperazine, the CF3, and electron-withdrawing groups have positive effects [80].

An open-label phase IIa trial to determine the EBA, extended EBA, and pharmacokinetics of LL-3858 for the treatment of newly diagnosed sputum smear-positive pulmonary TB has been completed, but the results are not available to the public [233]. Noteworthy, preliminary data suggest that LL-3858 has potent in vitro activity, with a MIC range of 0.06–0.5 μg/mL against Mtb, including MDR strains [234]. In combination with currently used antitubercular drugs, LL-3858 displays improved bacterial Cl in the lungs and spleen of the infected mice in lesser time than the conventional therapy. This was supported by another study where 12.5 mg/kg of LL-3858 reduced the mycobacterial load in mice to a greater extent than isoniazid [234]. Furthermore, van den Boogaard and co-workers demonstrated that LL-3858 exhibits concentration-dependent activity, without relapse observed up to 2 months following the final dose [234]. There is no reported information regarding efficacy in humans, although clinical trials might still be in progress in this respect.

### 2.11. InhA Inhibitors

**Figure 11:**
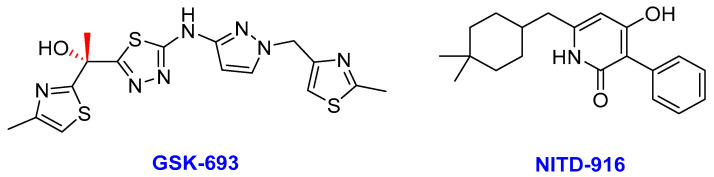
The chemical structures of GSK-693 and NITD-916; novel direct Mtb InhA inhibitors.

Enoyl-acyl carrier protein reductase (InhA) is a key enzyme in the type II fatty acid biosynthesis pathway (FASII) in Mtb. InhA was first identified as a clinical target based on the therapeutic potential of isoniazid in TB treatment [235,236]. InhA catalyzes the NADH-dependent reduction in long-chain trans-2-enoyl-acyl carrier proteins (ACPs) [235]. Isoniazid, an essential component of the TB drug regimens, targets the InhA of Mtb, and it is a prodrug activated by KatG—a mycobacterial catalase-peroxidase enzyme [237,238]. The dependency of isoniazid on KatG activation is the main clinical weakness (particularly drug resistance) associated with its application due to mutation occurrences in katG [239]. Thus, identifying inhibitors that directly bind to InhA without the requirement for activation by KatG (direct InhA inhibitors) may represent a valid strategy to overcome isoniazid resistance in TB treatment [240]. GSK-693 and NITD-916 (Figure 11) are novel direct InhA inhibitors of Mtb currently studied as potential substitutes for isoniazid in current TB treatment regimens.

(i) GSK-693

Lead optimization studies by GlaxoSmithKline (GSK) led to the identification of the thiadiazole chiral compound GSK-693 as a novel, selective, and promising lead compound with attractive antitubercular properties that overcome isoniazid resistance. GSK and other research units have carried out high-throughput screening against InhA using a collection of GSK compounds and identified the thiadiazole series to be the most promising compound class [241]. Despite the interesting in vitro antitubercular profile obtained from the initial SAR studies, hits were affected by a number of compound development requirements such as physicochemical properties, drug metabolism, and pharmacokinetics (DMPK) profiles [241].

Further tests confirmed that GSK-693 does not require KatG activation and retain the antitubercular activity against DS, MDR, and XDR clinical isolates [241]. Like other InhA direct inhibitors, thiadiazoles bind to the enzyme–NADH complex, but they do not establish any direct interaction with active site residue Tyr158 [242]. Unlike the isoniazid-NADH adduct, GSK-693 does not cause the flipping of the Phe149 side chain, and there is no interaction with the isonicotinic acid-binding pocket [241]. In addition, compounds inhibiting InhA without requiring activation by KatG could be active under anaerobic conditions where catalase-mediated activation is suppressed by the lack of oxygen.

GSK-693 displayed equal potency (MICs of 0.2 µg/mL) against the Mtb H37Rv inside and outside of macrophages, along with a good CYP 3A4 inhibition profile [241]. The compound demonstrates decreased lipophilicity, enhanced solubility, and reduced metabolic liabilities coupled with simultaneous good oral bioavailability at different doses in acute and chronic mouse models [165]. Lastly, early safety assessments done across different enzymatic assays indicated that GSK-693 did not show any sign of cytotoxicity and no hERG potassium channel inhibition [243], suggesting a low risk for general toxicity and cardiotoxicity observed in isoniazid.

(ii) NITD-916

More efforts to overcome isoniazid resistance in Mtb led to the discovery of a new class of direct InhA inhibitors called 4-hydroxy-2-pyridones, identified using phenotypic high-throughput screening [241,244]. The lead compound of the 4-hydroxy-2-pyridones series known as NITD-916 exhibits good activity against isoniazid-resistant MDR-TB clinical isolates and displays good efficacy in vivo in acute and established mouse TB infection models [95]. To inhibit InhA, NITD-916 forms a ternary complex with InhA-NADH, which blocks access to the enoyl substrate-binding site with a consequential reduction in the biosynthesis of mycolic acids and eventually cell death [245,246]. Based on the co-crystal structure, the 4-hydroxy-2-pyridones (including NITD-916) interact with the InhA–NADH complex through hydrogen bonding, *pi*-stacking, and hydrophobic interactions [247]. However, this mechanism of InhA inhibition was recently challenged by Flint et al. (2020) using killing kinetic studies to suggest that inhibition of InhA is bactericidal against nutrient-starved non-replicating Mtb, regardless of the binding mechanism [248]. This corroborates earlier findings that isoniazid reduces CFU in starved bacteria [249,250].

NITD-916 displays superior MIC against Mtb H37Rv over isoniazid (0.08 µg/mL vs. 0.8 µg/mL respectively) [251]; and also proves to have a lower frequency of resistance (1 × 10^−8^) than isoniazid (1 × 10^−5^) in vitro [95]. The difference in frequency of resistance is because isoniazid is a prodrug, and mutations in the KatG enzyme occur at a high frequency [251]. To this effect, NITD-916 overcomes a key liability associated with the application of isoniazid in current TB treatment regimens [251]. Thus, portraying potential as a novel TB therapeutic agent that is useful in guiding the development of improved hydroxy-pyridines and other direct InhA inhibitors.

### 2.12. β-Lactams

**Figure 12:**
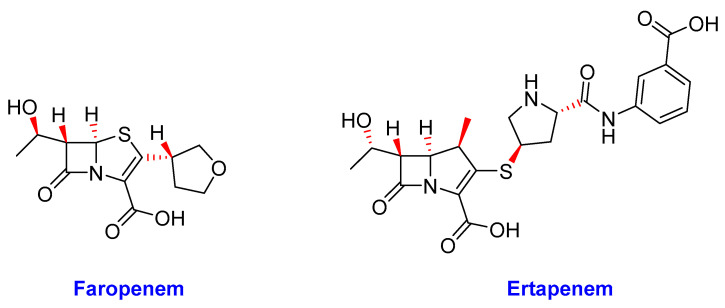
The chemical structures of faropenem and ertapenem.

The β-lactams are the most populous class of antibiotics and are the mainstay of most antibacterial drug treatment regimens; however, their efficacy against Mtb has always been limited [72]. Nonetheless, β-lactams generally offer a well-defined safety profile than other second-line alternatives [252]. Therefore, β-lactams: faropenem and ertapenem (Figure 12) briefly discussed below are being studied in phase II clinical trials [37].

(i) Faropenem

Faropenem is an orally bioavailable (72–84%) penem antibiotic that is more resistant to hydrolysis by β-lactamases than cephalosporins and carbapenems [253]. It is structurally similar to the carbapenems; however, this penem ring system is slightly less strained and consequently has improved chemical stability [254]. Additionally, it has been modified to a prodrug ester (faropenem medoxomil), which permits oral administration, both of which are desirable advantages for treating MDR-TB [72]. The drug is already approved for the treatment of respiratory infections in humans, but it also displayed promising bactericidal activity in both active and non-replicating Mtb cells comparable to meropenem [72,253].

Faropenem demonstrates potent biochemical activity independent of clavulanate (β-lactamase inhibitor) and inactivate Mtb L,D-transpeptidase enzymes more efficiently than meropenem [253]. Thus, the drug inhibits the L,D-transpeptidases, which perform the last cross-linking step of peptidoglycan synthesis. The MIC of faropenem against Mtb is 1.3 µg/mL with or without clavulanate, as opposed to an 8-fold increase in MIC for meropenem and many folds for other β-lactams tested in the absence of clavulanate [253]. Whilst using the dehydropeptidase inhibitor, probenecid; Dhar and colleagues demonstrated that Mtb-infected mice had a small but significant reduction in lung burden (7.7 and 7.5 log_10_ CFU/mouse at the beginning and end of treatment, respectively) after 9 days of treatment with a combination of faropenem/clavulanate/probenecid [253,255].

In summary, faropenem’s stability, oral bioavailability, superior biochemical potency against its molecular target, and proven efficacy in the treatment of infections caused by nontuberculous mycobacteria all justify its potential in the chemotherapy of MDR-TB and XDR-TB [256].

(ii) Ertapenem

The second β-lactam agent to have shown promising clinical results and favorable pharmacokinetic properties in the treatment of Mtb is ertapenem, a member of the carbapenem class of antibiotics [151]. Amongst all β-lactams, the carbapenems are the most suitable for treating Mtb as they potently target the high molecular weight penicillin-binding proteins and also inactivate the unusual L,D-transpeptidases that form the 3→3 crosslinks found in the unique Mtb cell wall [253,257]. Carbapenems inhibit the peptidase domain of penicillin-binding proteins, leading to autolysis and peptidoglycan weakening of the cell wall [258]. The development of carbapenems for TB treatment has raised considerable interest recently because the presence of clavulanic acid establishes uniform activity against XDR-Mtb and kills both growing and dormant forms of the bacilli [258]. The carbapenems are less favorable substrates for the Mtb β-lactamase BlaC due to rapid acylation and slow deacylation [72]. As such, unlike other β-lactams, they are not rapidly hydrolyzed by BLaC and thus maintain their activity against Mtb [259]. Although encouraging results have recently been obtained using meropenem for the treatment of XDR-TB in humans, the compound is unstable and has poor bioavailability [253]. As a result, frequent intravenous administrations of the drug are required, making its general clinical application limited [165].

Following reconstitution and dilution, Ertapenem degradation is temperature-dependent. Thus, the proposed in-use shelf life for ertapenem is 6 h at room temperature, 24 h at 2–8 °C [151]. Since Mtb has a doubling time of at least 24 h under the best of circumstances [260], microbial killing and inhibition of growth by the most effective of antibiotics, especially by β-lactams, are slow (requiring several days) because the drugs depend on cell wall turnover [151]. Consequently, drugs such as ertapenem, already appearing unstable at 37 °C [259], are likely to be degraded before killing or inhibiting slow-growing bacteria, especially semi-dormant Mtb. However, drug susceptibility tests show that ertapenem is likely to have a good sterilizing effect in TB [151]. This might be attributed to its rapid inactivation of L,D-transpeptidases (which is more rapid than imipenem and meropenem), as well as due to its reversible protein binding nature [257]. To reach maximal bactericidal activity for Gram-positive, Gram-negative, and anaerobic bacterial infections, ertapenem is given intravenously at a dose of 1000 mg once daily [261]. Ertapenem has a long plasma half-life of 4 h, compared to other carbapenems, enabling once-daily dosing, which is an advantage for MDR-TB treatment [262,263]. The AUC_0–24_ of ertapenem is 544.9 h·mg/L with a Cmax of 127.5 mg/L in MDR-TB patients [259].

Generally, ertapenem treatment is well tolerated during MDR-TB treatment [259,264]. There are no drug-drug interactions, and the drug is not metabolized by cytochrome P450, nor is it a substrate for p-glycoprotein [265,266]. Renal excretion is the main route of elimination for ertapenem. Therefore, the pharmacokinetics of the drug are altered to a significant extent clinically in patients with severe renal impairment [267]. In such cases, a dose reduction to 500 mg once a day is recommended [266].

### 2.13. Oxoborates

Recently, boron-containing compounds known as oxaboroles have been shown to inhibit LeuRS (Leucyl-tRNA synthetase) by the oxaborole tRNA-trapping mechanism [268]. The aminoacyl-tRNA synthetases are a family of essential enzymes that are required for protein synthesis in all cells [269]. The boron atom is an integral part of Mtb LeuRS inhibitors, as it forms a bidentate covalent adduct with the terminal adenosine nucleotide Ade76 of tRNALeu [269]. Consequently, this covalent adduct traps the 3′ end of the tRNALeu in the editing site forming a nonproductive complex, blocking leucylation and bacterial protein synthesis [268].

(i) GSK-3036656 (GSK-070)

**Figure 13:**
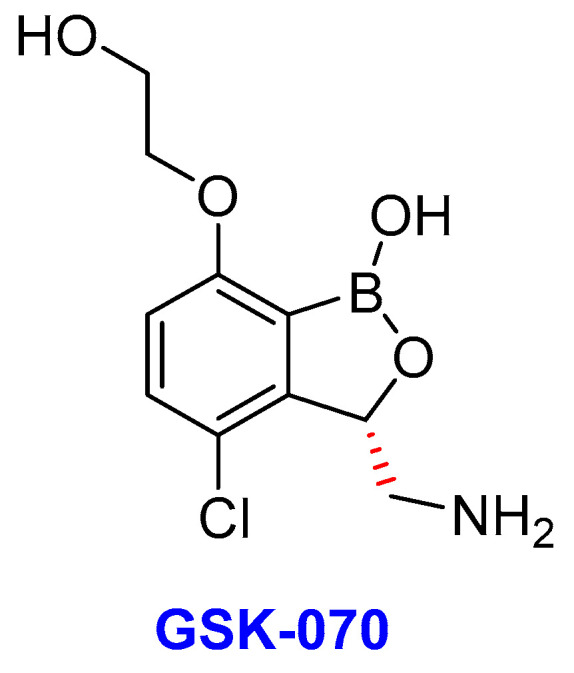
The chemical structure of GSK-070, an oxoborate drug in TB clinical trials.

Another product of potential interest discovered by the GSK group is GSK-3036656, alternatively known as GSK-070 (Figure 13) is currently in a phase II clinical trial for the treatment of TB [270]. GSK-070 is a novel 3-aminomethyl-4-chloro-benzoxaborole with inhibitory activity against the Mtb enzyme LeuRS [271]. Thus, GSK-070 binds to the catalytic site of LeuRS that is responsible for the hydrolysis of incorrectly ligated aminoacylated tRNAs [179]. Notably, the binding is necessitated by the presence of the amino group of the (S)-aminomethyl side chain at C-3 through hydrogen-bonding interactions [271]. Whereas the halogen substituents (Cl or Br) improves the Mtb’s LeuRS and antitubercular activity against Mtb H37Rv as well as selectivity against other bacteria [272]. GSK-070 exhibited promising activity against laboratory strains of Mtb as well as against selected DS-TB, MDR-TB, and XDR-TB clinical isolates [271].

The efficacy of GSK-070 was assessed against replicating and nonreplicating/slow-replicating mycobacteria in mice models of acute and chronic Mtb H37Rv lung infections [273,274]. In the acute model, a maximum difference of 3.6 log_10_ CFU was obtained in the lungs compared to untreated mice for 8 days; whereas the chronic cohort produced a drop of 2.1 log_10_ CFU after 2 months of daily oral treatment at maximum doses of 1.1 and 1.3 mg/kg, respectively [273]. A recent phase 1 clinical study evaluating the safety, tolerability, and pharmacokinetics of single and repeat oral doses of GSK-070 met all primary endpoints and showed no serious adverse events [179,275]. The study determined potent inhibition of Mtb LeuRS (IC_50_ = 0.20 µM) and in vitro antitubercular activity (Mtb H37Rv MIC = 0.08 µg/mL) [272]. GSK-070 showed a dose-proportional increase following single-dose administration after dosing for 14 days. Additionally, the drug showed 2–3 fold increase in Cmax and AUC accumulations with repeated administration after the dosing period that was unaltered in the presence of food [271]. Furthermore, GSK-070 is a low-hepatic-clearance compound with high solubility and moderate passive permeability and is not a p-glycoprotein substrate; hence it undergoes rapid absorption and exhibited very high oral bioavailability in the nonclinical species (mouse, rat, and dog) [271]. Finally, in vitro data suggest that GSK-070 has a low plasma protein binding in the nonclinical species (mice, rats, and dogs, and humans) [271]. The main route of elimination for GSK-070 is in the urine.

Table 1 below summarizes the mutation event(s) associated with resistance against novel anti-TB drugs and drug candidates.

## 3. Conclusions

To date, effective antibiotics for TB treatment have been discovered through tireless efforts from drug development industries. However, the current treatment regimens require a very long treatment period to achieve an effective cure, and they are made up of several drugs, some of which may not be well tolerated. The long treatment period and therapeutic side effects lead to patients’ non-compliance to treatment, causing increased selection of resistant mutants. Therefore, TB remains a global health care problem that affects over ten million people and kills at least a million each year. Specifically, the increasing prevalence of MDR- and XDR-TB is worrisome, hence the need to continuously engage in the development of new drug regimens against TB.

To this effect, studies to optimize the dose and treatment duration of currently licensed drugs show successful preliminary results. However, discoveries of repurposed drugs and novel chemotherapeutic regimens that are of less toxicity, have high efficacy, and achieve effective cure in less than two months are much anticipated. These can be achieved by understanding the resistance mechanism(s) of new TB drugs, fostering synthesis of target-based chemical libraries with whole-cell activity and high-throughput screening to identify novel agents, as well as identification of agents with activity against the slow replicating forms of Mtb. Additionally, prevention of TB transmission with an effective vaccine when possible will hopefully help in eradicating this global burden. Therefore, the combination of effective vaccines, immunotherapy, and new drugs may contribute to shortening the treatment period as well as simplify the regimen.

Combination therapy, for sure, will remain the gold standard for TB treatment and the treatment of TB and co-morbidities such as diabetes, HIV infection; it is, therefore, pertinent to establish the pharmacokinetic properties of new TB drug candidates as well as know the pharmacokinetic properties of existing drugs. This should enable the selection of a drug combination wherein the individual drugs have complimentary pharmacokinetics properties, hence avoiding monotherapy at the target site after drug administration. Another pertinent issue in combination therapy and treatment of co-morbidities is the effect of drug substrates on cytochrome P450 isoenzymes, which often lead to treatment failure and/or contraindication. The effect of new TB drug candidates on cytochrome P450 isoenzymes should be established and compared against other TB drugs (candidates) as well as drugs used against common co-morbidities to enable the selection of ideal combination therapies void of drug-drug interactions.

So far, the TB Alliance, a non-profit organization, has championed the development of several drug combinations with the aim of identifying simpler regimens consisting of fewer drugs that can achieve a complete cure in two months or less.

Table 2 below summarizes the compounds currently in development as potential drugs for TB treatment under respective chemical classes, the development stage (lead optimization, pre-clinical or advanced trial) as well as their biological targets, and reported pharmacokinetic data.

## Figures and Tables

**Table 1:** Mutant genes and Mutations causing Mtb resistance to novel anti-TB drugs under development.

Drug	Mutant Gene	Mutation(s)	References
MXF	gyrA	D94G, D94N, and D94Y	Nosova et al. 2013; Groll et al. 2009
GFX	gyrA gyrB	A90V, A94G, A94T, A94A, A94H and A89A Δ678, Δ679, and A533T	Nosova et al. 2013; Groll et al. 2009
BDQ	atpE rv0678	A63P, A63V, D28A, D28V, D28P, D28N, D28G, R124stop, L40F, T91P, and E21stop G66E, M1A, W42R, S53L, S53P, S63R, and S63G	Huitric et al. 2010; Segala et al. 2012; Zimenkov et al. 2017; Andries et al. 2014; Pang et al. 2017; Xu et al. 2018; Zhang et al. 2015
LZD	rplC rrl	C154R G2299T, G2814T, G2270T, and G2746A	McNeil et al. 2017; Zhang et al. 2016; Pang 2017; Balasubramanian et al. 2014, Zhang et al. 2014; McNeil et al. 2017
DM	ddn fgd1	L107P and 59–101 (deletion) T960C	Schena et al. 2016, Fujiwara et al. 2017
PM	ddn fgdi fbiC	V616, Y89H, and Y133D R212Q A2158A and C1114T	Haver et al. 2015
SQ-109	mmPL3	A700T, L567P, Q40R, and T2055375C	Tahlan et al. 2012
BTZ-043	dprE1	C387G, C387S, C387A, C387T, and C387N	Foo et al. 2016
PBTZ-169	dprE1	C387G, C387S, C387A, C387T, and C387N	Foo et al. 2016; Chen et al. 2020
OPC-167832	dprE1 (rv3790) mmpS5-mmpL5 (rv0678)	C387G, C387S, C387A, C387T, C387N, and V388T G248C, A364S,T314H, and 84–85InsIS6110	Hariguchi et al. 2020; Milano et al 2009
TBA-7371	dprE1	Y314H	Gawad and Bonde 2018
Q203	qcrB	T313A	
NITD-916	inhA fabG1InhA	S19W, I21M, I21V, F41L, F47L, S94A, M103T, D148E, M161L, R195G, I202F, G205S, G205A, G205R, A206E, G212D, G214P, I215S, L269R, Δ210, T162M, and R49H C-15T	McNeil 2017

**Table 2:** Anti-TB drug candidates in different phases of the drug development process: 2010–2020.

Chemical Class	Novel Drug	MW g/mol	LogP (ChemDraw)	Clinical Phase	Cellular Target	T_max_ h	AUC mg.h/L ^a^ ng.h/mL ^b^ µg.h/mL^c^	T_1/2_ h	CYP450 Inhibition
Fluoroquinolones	Moxifloxacin	401.43	1.60	III	DNA gyrase and Topoisomerases	1.5	26.9^a^	11.5–15.6	–
	Gatifloxacin	375.39	1.2	III	DNA gyrase and Topoisomerases	1–2	51.3 ^a^	7–14	–
	DC-195a	421.44	1.24	Pre-clinical	DNA Replication	–	15.8 ^a^	1.51–1.93	Un-confirmed
Diarylquinolones	Bedaquiline	555.50	7.52	IIb/III	ATP Synthase	4–6	65 ^a^	21.7–24	Moderate–high risk
	TBAJ-876	657.56	6.08	Pre-clinical	ATP Synthase	–	4.61 ^c^	–	–
	TBAJ-587	614.5	6.4	Pre-clinical	ATP Synthase	–	1.72 ^c^	–	–
Oxazolidinone	Linezolid	337.35	0.58	Iib	Protein synthesis	1–2	210 ^a^	6–7.9	–
	Sutezolid	353.41	1.3	Iia	Protein synthesis	0.5	31945 ^b^	2.8–4	Substrate
	AZD5847	465.40	0.7	Iia	Protein synthesis	2–4	93.19 ^c^	7–11	–
Nitroimidazoles	Delamanid	534.5	—	III	Cell Wall Acids Synthesis	4–5	2.9 ^a^	30–38	Liver CYP3A
	Pretomanid	359.26	—	III	Cell Wall, Lipids, and Protein synthesis	4–5	53 ^c^	10–30	Insignificant
	TBA 354	436.30	—	II	Cell Wall, Lipids, and Protein synthesis	2–6	22.7–242 ^c^	8–12	Weak CYP3A4
Ethylenediamines	SQ109	330.50	4.44	Iib/III	Cell Wall Acids Synthesis	1	183.7–268.5 ^b^	19.6	CYP_2_D_6_, CYP_2_C_19_ & weak CYP3A4
Benzothiazinones	BTZ043	431.40	—	Pre-clinical	DprE1	0.25	899 ^b^	1.22	Low
	PBTZ169	456.48	4.42	II	DprE1	1.5–2.5	5478 ^b^	2.87	–
Carbostyrils	OPC-167832	456.85	2.82	I	DprE1	0.5–1	Dose dependent	1.3–2.1	–
	TBA-7371	355.40	1.31	I	DprE1	–	166–240 ^c^	–	–
Imidazopyridine amides	Q203	557.01	7.64	Pre-clinical	QcrB	2	44100 ^b^	23.4	–
	TB47	538.57	6.61		QcrB	3.2	33144 ^c^	35.6	Insignificant
	ND-11543	532.56	5.84		QcrB	2	11704 ^b^	24	Substrate
Caprazamycins	CPZEN-45	688.70	−2.59	Pre-clinical	Cell wall Peptidoglycan biosynthesis (WecA)	–	–	–	–
Oxaboroles	GSK070 (3036656)	257.48	—	II	LeuRS	–	–	–	–
Riminophenazines	TBI-166	589.60	5.08	Pre-clinical	DNA Synthesis (QcrB)	2.7	2658.2 ^b^	20.4	–
Hydrazides	LL3858	519.57	5.31	I	Undefined	–	–	–	–
Pyrroles	BM212	414.40	5.30	Lead Optimisation	MmpL3 Protein	–	–	–	–
Oxoborates	GSK-693	419.54	5.69	Lead Optimisation	InhA	–	–	–	CYP3A4
	NITD-916	311.42	2.56	Lead Optimisation	InhA	–	–	–	–
Β-lactams	Faropenem	285.31	−1.62	II	L,D-traspeptidase	2	16.2 ^a^	1.2	–
	Ertapenem	475.52	−1.72	II	L,D-traspeptidase	–	544.9 ^a^	4	–

Units for AUC: ^a^ = mg.h/L, ^b^ = ng.h/mL, ^c^ = µg.h/mL.

## Data Availability

The study did not include data analysis.

## References

[1] Patil J. (2015). Novel tubercular therapeutic agents: Need of the Day. Pharmacoepidemiol. Drug Saf..

[2] World Health Organization. https://www.who.int/teams/global-tuberculosis-programme/tb-reports/global-report-2019.

[3] Brites D., Loiseau C., Menardo F., Borrell S., Boniotti M., Warren R., Dippenaar A., Parsons S.D., Beisel C., Behr M.A. (2018). A new phylogenetic framework for the animal-adapted Mycobacterium tuberculosis complex. Front. Microbiol..

[4] Gutierrez M.C., Brisse S., Brosch R., Fabre M., Omaïs B., Marmiesse M., Supply P., Vincent V. (2005). Ancient origin and gene mosaicism of the progenitor of Mycobacterium tuberculosis. PLoS Pathog..

[5] Glaziou P., Sismanidis C., Floyd K., Raviglione M. (2014). Global epidemiology of tuberculosis. Cold Spring Harb Perspect. Med..

[6] Lin P.L., Flynn J.L. (2010). Understanding latent tuberculosis: A moving target. J. Immunol..

[7] Heemskerk D., Caws M., Marais B., Farrar J. (2015). Tuberculosis in Adults and Children.

[8] Moghadam M.T., Amirmozafari N., Shariati A., Hallajzadeh M., Mirkalantari S., Khoshbayan A., Jazi F.M. (2020). How phages overcome the challenges of drug resistant bacteria in clinical infections. Infect. Drug Resist..

[9] Castañeda-Hernández D.M., Rodriguez-Morales A.J., Rodriguez-Morales A.J. (2013). Epidemiological burden of tuberculosis in developing countries. Current Topics in Public Health.

[10] Kawatsu L., Uchimura K., Izumi K., Ohkado A., Ishikawa N. (2016). Profile of tuberculosis among the foreign-born population in Japan, 2007–2014. Western Pac. Surveill Response J..

[11] Sakamoto H., Lee S., Ishizuka A., Hinoshita E., Hori H., Ishibashi N., Komada K., Norizuki M., Katsuma Y., Akashi H. (2019). Challenges and opportunities for eliminating tuberculosis–leveraging political momentum of the UN high-level meeting on tuberculosis. BMC Public Health.

[12] Kumar K., Kon O.M. (2017). Diagnosis and treatment of tuberculosis: Latest developments and future priorities. Ann. Res. Hosp..

[13] Patil J. (2014). New theoretical background for tuberculosis treatment. J. Pharmacovigil..

[14] Kanabus A. (2020). Information about Tuberculosis. GHE: United Kingdom. https://www.tbfacts.org.

[15] Eker B., Ortmann J., Migliori G.B., Sotgiu G., Muetterlein R., Centis R., Hoffmann H., Kirsten D., Schaberg T., Ruesch-Gerdes S. (2008). Multidrug-and extensively drug-resistant tuberculosis, Germany. Emerg. Infect. Dis..

[16] World Health Organisation. https://www.who.int/tb/publications/2019/consolidated-guidelines-drug-resistant-TB-treatment/en/.

[17] Sharma S.K., Dheda K. (2019). What is new in the WHO consolidated guidelines on drug-resistant tuberculosis treatment?. Indian J. Med. Res..

[18] Rendon A., Tiberi S., Scardigli A., D’Ambrosio L., Centis R., Caminero J.A., Migliori G.B. (2016). Classification of drugs to treat multidrug-resistant tuberculosis (MDR-TB): Evidence and perspectives. J. Thorac. Dis..

[19] Brown T.S., Challagundla L., Baugh E.H., Omar S.V., Mustaev A., Auld S.C., Shah N.S., Kreiswirth B.N., Brust J.C., Nelson K.N. (2019). Pre-detection history of extensively drug-resistant tuberculosis in KwaZulu-Natal, South Africa. Proc. Natl. Acad. Sci. USA..

[20] Nguyen L. (2016). Antibiotic resistance mechanisms in M. tuberculosis: An update. Arch. Toxicol..

[21] Smith T., Wolff K.A., Nguyen L., Pieters J., McKinney J. (2012). Molecular biology of drug resistance in Mycobacterium tuberculosis. Pathogenesis of Mycobacterium Tuberculosis and Its Interaction with the Host Organism.

[22] Arya N., Raut M.K., Tekale S.G., Shishoo C.J., Jain K.S. (2014). Tuberculosis: New drug discovery pipelines. Austin J. Anal. Pharm. Chem..

[23] Poissy J., Aubry A., Fernandez C., Lott M.-C., Chauffour A., Jarlier V., Farinotti R., Veziris N. (2010). Should moxifloxacin be used for the treatment of extensively drug-resistant tuberculosis? An answer from a murine model. Antimicrob. Agents Chemother..

[24] Aubry A., Pan X.-S., Fisher L.M., Jarlier V., Cambau E. (2004). Mycobacterium tuberculosis DNA gyrase: Interaction with quinolones and correlation with antimycobacterial drug activity. Antimicrob. Agents Chemother..

[25] Madhusudan K., Ramesh V., Nagaraja V. (1994). Molecular cloning of gyrA and gyrB genes of Mycobacterium tuberculosis: Analysis of nucleotide sequence. Int. J. Biochem. Mol. Biol..

[26] Gillespie S.H. (2016). The role of moxifloxacin in tuberculosis therapy. Eur. Respir. J..

[27] Jindani A., Harrison T.S., Nunn A.J., Phillips P.P., Churchyard G.J., Charalambous S., Hatherill M., Geldenhuys H., McIlleron H.M., Zvada S.P. (2014). High-dose rifapentine with moxifloxacin for pulmonary tuberculosis. N. Engl. J. Med..

[28] Naidoo A., Naidoo K., McIlleron H., Essack S., Padayatchi N. (2017). A review of moxifloxacin for the treatment of drug-susceptible tuberculosis. J. Clin. Pharmacol..

[29] Dookie N., Sturm A.W., Moodley P. (2014). Moxifloxacin resistance in the F15/LAM4/KZN extensively drug-resistant strain of Mycobacterium tuberculosis. Infect. Drug Resist..

[30] Pletz M.W.R., De Roux A., Roth A., Neumann K.-H., Mauch H., Lode H. (2004). Early bactericidal activity of moxifloxacin in treatment of pulmonary tuberculosis: A prospective, randomized study. Antimicrob. Agents Chemother..

[31] Rodríguez J.C., Ruiz M., López M., Royo G. (2002). In vitro activity of moxifloxacin, levofloxacin, gatifloxacin and linezolid against Mycobacterium tuberculosis. Int. J. Antimicrob. Agents.

[32] Balfour J.A.B., Wiseman L.R. (1999). Moxifloxacin. Drugs.

[33] Nijland H., Ruslami R., Suroto A.J., Burger D., Alisjahbana B., Van Crevel R., Aarnoutse R.E. (2007). Rifampicin reduces plasma concentrations of moxifloxacin in patients with tuberculosis. Clin. Infect. Dis..

[34] Al Omari M.M., Jaafari D.S., Al-Sou’od K.A., Badwan A.A. (2014). Moxifloxacin hydrochloride. Profiles Drug Subst. Excip. Relat. Methodol..

[35] Zvada S.P., Denti P., Sirgel F.A., Chigutsa E., Hatherill M., Charalambous S., Mungofa S., Wiesner L., Simonsson U.S., Jindani A. (2014). Moxifloxacin population pharmacokinetics and model-based comparison of efficacy between moxifloxacin and ofloxacin in African patients. Antimicrob. Agents Chemother..

[36] Grossman R.F., Hsueh P.-R., Gillespie S.H., Blasi F. (2014). Community-acquired pneumonia and tuberculosis: Differential diagnosis and the use of fluoroquinolones. Int. J. Infect. Dis..

[37] Hofman S., Segers M., Ghimire S., Bolhuis M., Sturkenboom M., Van Soolingen D., Alffenaar J.W. (2016). Emerging drugs and alternative possibilities in the treatment of tuberculosis. Expert Opin. Emerg. Drugs.

[38] Gajjar D.A., LaCreta F.P., Uderman H.D., Kollia G.D., Duncan G., Birkhofer M.J., Grasela D.M. (2000). A dose-escalation study of the safety, tolerability, and pharmacokinetics of intravenous gatifloxacin in healthy adult men. Pharmacotherapy.

[39] Nakashima M., Uematsu T., Kosuge K., Kusajima H., Ooie T., Masuda Y., Ishida R.Y., Uchida H.I. (1995). Single-and multiple-dose pharmacokinetics of AM-1155, a new 6-fluoro-8-methoxy quinolone, in humans. Antimicrob. Agents Chemother..

[40] Deshpande D., Pasipanodya J.G., Srivastava S., Bendet P., Koeuth T., Bhavnani S.M., Ambrose P.G., Smythe W., McIlleron H., Thwaites G. (2018). Gatifloxacin pharmacokinetics/pharmacodynamics–based optimal dosing for pulmonary and meningeal multidrug-resistant tuberculosis. Clin. Infect. Dis..

[41] LaCreta F.P., Kaul S., Kollia G.D., Duncan G., Randall D.M., Grasela D.M. (2000). Interchangeability of 400-mg intravenous and oral gatifloxacin in healthy adults. Pharmacotherapy.

[42] Grasela D.M. (2000). Clinical pharmacology of gatifloxacin, a new fluoroquinolone. Clin. Infect. Dis..

[43] Mignot A., Guillaume M., Brault M., Gualano V., Millérioux L., Göhler K., Stahlberg H.J. (2002). Multiple-dose pharmacokinetics and excretion balance of gatifloxacin, a new fluoroquinolone antibiotic, following oral administration to healthy Caucasian volunteers. Chemotherapy.

[44] Sarkar S., Ganguly A., Sunwoo H. (2016). Current overview of anti-tuberculosis Drugs: Metabolism and toxicities. Mycobact. Dis..

[45] Chiang C., Van Deun A., Rieder H. (2016). Gatifloxacin for short, effective treatment of multidrug-resistant tuberculosis. Int. J. Tuberc. Lung. Dis..

[46] Quan D., Nagalingam G., Payne R., Triccas J.A. (2017). New tuberculosis drug leads from naturally occurring compounds. J. Infect. Dis..

[47] Ahmad Z., Minkowski A., Peloquin C.A., Williams K.N., Mdluli K.E., Grosset J.H., Nuermberger E.L. (2011). Activity of the fluoroquinolone DC-159a in the initial and continuation phases of treatment of murine tuberculosis. Antimicrob. Agents Chemother..

[48] Onodera Y., Hirata T., Hoshino K., Otani T. DC-159a, a novel quinolone, showed high inhibitory activity against altered topoisomerases of Streptococcus pneumoniae and Mycobacterium tuberculosis. Proceedings of the 47th Interscience Conference on Antimicrobial Agents and Chemotherapy.

[49] Asif M., Farhan S.S. (2020). An overview on antitubercular activity profile of fluoroquinolone derivatives and their molecular hybridization. J. Med. Chem..

[50] Disratthakit A., Doi N. (2010). In vitro activities of DC-159a, a novel fluoroquinolone, against Mycobacterium species. Antimicrob. Agents Chemother..

[51] Sekiguchi J., Disratthakit A., Maeda S., Doi N. (2011). Characteristic resistance mechanism of Mycobacterium tuberculosis to DC-159a, a new respiratory quinolone. Antimicrob. Agents Chemother..

[52] Villemagne B., Crauste C., Flipo M., Baulard A.R., Déprez B., Willand N. (2012). Tuberculosis: The drug development pipeline at a glance. Eur. J. Med. Chem..

[53] Mdluli K., Ma Z. (2007). Mycobacterium tuberculosis DNA gyrase as a target for drug discovery. Infect. Disord. Drug Targets..

[54] Schaaf H.S., Thee S., van der Laan L., Hesseling A.C., Garcia-Prats A.J. (2016). Adverse effects of oral second-line antituberculosis drugs in children. Expert Opin. Drug Saf..

[55] Koul A., Vranckx L., Dendouga N., Balemans W., Van den Wyngaert I., Vergauwen K., Göhlmann H.W., Willebrords R., Poncelet A., Guillemont J. (2008). Diarylquinolines are bactericidal for dormant mycobacteria as a result of disturbed ATP homeostasis. J. Biol. Chem..

[56] Biuković G., Basak S., Manimekalai M.S.S., Rishikesan S., Roessle M., Dick T., Rao S.P., Hunke C., Grüber G. (2013). Variations of subunit ε of the Mycobacterium tuberculosis F1Fo ATP synthase and a novel model for mechanism of action of the tuberculosis drug TMC207. Antimicrob. Agents Chemother..

[57] Olaru I.D., von Groote-Bidlingmaier F., Heyckendorf J., Yew W.W., Lange C., Chang K.C. (2015). Novel drugs against tuberculosis: A clinician’s perspective. Eur. Respir. J..

[58] Hards K., McMillan D.G., Schurig-Briccio L.A., Gennis R.B., Lill H., Bald D., Cook G.M. (2018). Ionophoric effects of the antitubercular drug bedaquiline. Proc. Natl. Acad. Sci. USA.

[59] Matteelli A., Carvalho A.C., Dooley K.E., Kritski A. (2010). TMC207: The first compound of a new class of potent anti-tuberculosis drugs. Future Microbiol..

[60] Dooley K.E., Park J.-G., Swindells S., Allen R., Haas D.W., Cramer Y., Aweeka F., Wiggins I., Gupta A., Lizak P. (2012). Safety, tolerability, and pharmacokinetic interactions of the antituberculous agent TMC207 (bedaquiline) with efavirenz in healthy volunteers: AIDS Clinical Trials Group Study A5267. J. Acquir. Immune Defic. Syndr..

[61] Pethe K., Bifani P., Jang J., Kang S., Park S., Ahn S., Jiricek J., Jung J., Jeon H.K., Cechetto J. (2013). Discovery of Q203, a potent clinical candidate for the treatment of tuberculosis. Nat. Med..

[62] Andries K., Verhasselt P., Guillemont J., Göhlmann H.W., Neefs J.-M., Winkler H., Van Gestel J., Timmerman P., Zhu M., Lee E. (2005). A diarylquinoline drug active on the ATP synthase of Mycobacterium tuberculosis. Science.

[63] Gobedo A., Hwoldi A., Toma A. (2015). Recent advances in the development of anti-tuberculosis drugs acting on multidrug-resistant strains: A review. Int. J. Pharm. Biol. Sci..

[64] Nguyen T., Cao T., Akkerman O., Tiberi S., Vu D., Alffenaar J. (2016). Bedaquiline as part of combination therapy in adults with pulmonary multi-drug resistant tuberculosis. Expert Rev. Clin. Pharmacol..

[65] Van Heeswijk R.P.G., Dannemann B., Hoetelmans R.M.W. (2014). Bedaquiline: A review of human pharmacokinetics and drug–drug interactions. Antimicrob. Agents Chemother..

[66] Lounis N., Gevers T., Van Den Berg J., Andries K. (2008). Impact of the interaction of R207910 with rifampin on the treatment of tuberculosis studied in the mouse model. Antimicrob. Agents Chemother..

[67] Tiberi S., du Plessis N., Walzl G., Vjecha M.J., Rao M., Ntoumi F., Mfinanga S., Kapata N., Mwaba P., McHugh T.D. (2018). Tuberculosis: Progress and advances in development of new drugs, treatment regimens, and host-directed therapies. Lancet Infect. Dis..

[68] Fox G.J., Menzies D. (2013). A review of the evidence for using bedaquiline (TMC207) to treat multi-drug resistant tuberculosis. Infect. Dis Ther..

[69] Van Heeswijk R., Vandevoorde A., Meyvisch P., De Marez T., De Beule K., Mc Neeley D., Hoetelmans R. The effect of lopinavir/ritonavir on the pharmacokinetics of TMC207, an investigational antimycobacterial agent. Proceedings of the 18th International AIDS Conference.

[70] Svensson E.M., Aweeka F., Park J.-G., Marzan F., Dooley K.E., Karlsson M.O. (2013). Model-based estimates of the effects of efavirenz on bedaquiline pharmacokinetics and suggested dose adjustments for patients coinfected with HIV and tuberculosis. Antimicrob. Agents Chemother..

[71] Coyne K.M., Pozniak A.L., Lamorde M., Boffito M. (2009). Pharmacology of second-line antituberculosis drugs and potential for interactions with antiretroviral agents. Aids.

[72] Hoagland D.T., Liu J., Lee R.B., Lee R.E. (2016). New agents for the treatment of drug-resistant Mycobacterium tuberculosis. Adv. Drug Deliv. Rev..

[73] Pontali E., Sotgiu G., Tiberi S., D’Ambrosio L., Centis R., Migliori G.B. (2017). Cardiac safety of bedaquiline: A systematic and critical analysis of the evidence. Eur. Respir. J..

[74] Sarathy J.P., Ragunathan P., Shin J., Cooper C.B., Upton A.M., Grüber G., Dick T. (2019). TBAJ-876 retains bedaquiline’s activity against subunits c and ε of Mycobacterium tuberculosis F-ATP synthase. Antimicrob. Agents Chemother..

[75] Sutherland H.S., Tong A.S., Choi P.J., Conole D., Blaser A., Franzblau S.G., Cooper C.B., Upton A.M., Lotlikar M.U., Denny W.A. (2018). Structure-activity relationships for analogs of the tuberculosis drug bedaquiline with the naphthalene unit replaced by bicyclic heterocycles. Bioorg. Med. Chem..

[76] Choi P.J., Sutherland H.S., Blaser A., Tong A.S., Cooper C.B., Upton A.M., Upton A.M., Palmer B.D., Denny W.A. (2020). Synthetic studies to help elucidate the metabolism of the preclinical candidate TBAJ-876—A less toxic and more potent analogue of bedaquiline. Molecules.

[77] Sarathy J.P., Gruber G., Dick T. (2019). Re-understanding the mechanisms of action of the anti-mycobacterial drug bedaquiline. Antibiotics.

[78] Sutherland H.S., Tong A.S., Choi P.J., Blaser A., Conole D., Franzblau S.G., Lotlikar M.U., Cooper C.B., Upton A.M., Denny W.A. (2019). 3, 5-Dialkoxypyridine analogues of bedaquiline are potent antituberculosis agents with minimal inhibition of the hERG channel. Bioorg. Med. Chem..

[79] Sutherland H.S., Tong A.S., Choi P.J., Blaser A., Franzblau S.G., Cooper C.B., Upton A.M., Lotlikar M., Denny W.A., Palmer B.D. (2020). Variations in the C-unit of bedaquiline provides analogues with improved biology and pharmacology. Bioorg. Med. Chem..

[80] Kumar D., Negi B., Rawat D.S. (2015). The anti-tuberculosis agents under development and the challenges ahead. Future Med. Chem..

[81] Kwon Y.-S., Jeong B.-H., Koh W.-J. (2015). Delamanid when other anti-tuberculosis-treatment regimens failed due to resistance or tolerability. Expert Opin. Pharmacother..

[82] Szumowski J.D., Lynch J.B. (2015). Profile of delamanid for the treatment of multidrug-resistant tuberculosis. Drug Des. Devel. Ther..

[83] Karekar S.R., Marathe P.A. (2018). Current status of delamanid in the management of MDR tuberculosis. J. Assoc. Physicians India.

[84] Ryan N.J., Lo J.H. (2014). Delamanid: First global approval. Drugs.

[85] TB Alliance. https://www.treatmentactiongroup.org/wp-content/uploads/2019/12/pipeline_tb_treatment_lm_final.pdf.

[86] Saliu O.Y., Crismale C., Schwander S.K., Wallis R.S. (2007). Bactericidal activity of OPC-67683 against drug-tolerant Mycobacterium tuberculosis. J. Antimicrob. Chemother..

[87] Xavier A.S., Lakshmanan M. (2014). Delamanid: A new armor in combating drug-resistant tuberculosis. J. Pharm. Pharm..

[88] Blair H.A., Scott L.J. (2015). Delamanid: A review of its use in patients with multidrug-resistant tuberculosis. Drugs.

[89] Shimokawa Y., Sasahara K., Yoda N., Mizuno K., Umehara K. (2014). Delamanid does not inhibit or induce cytochrome p450 enzymes in vitro. Biol. Pharm. Bull..

[90] Gler M.T., Skripconoka V., Sanchez-Garavito E., Xiao H., Cabrera-Rivero J.L., Vargas-Vasquez D.E., Gao M., Awad M., Park S.K., Shim T.S. (2012). Delamanid for multidrug-resistant pulmonary tuberculosis. N. Engl. J. Med..

[91] Smith M., Accinelli A., Tejada F., Kharel M. (2017). Drugs used in tuberculosis and leprosy. Side Effects of Drugs Annual. 39.

[92] Mallikaarjun S., Wells C., Petersen C., Paccaly A., Shoaf S.E., Patil S., Geiter L. (2016). Delamanid coadministered with antiretroviral drugs or antituberculosis drugs shows no clinically relevant drug-drug interactions in healthy subjects. Antimicrob. Agents Chemother..

[93] Lewis J.M., Sloan D.J. (2015). The role of delamanid in the treatment of drug-resistant tuberculosis. Clin. Risk Manag..

[94] Tyagi S., Nuermberger E., Yoshimatsu T., Williams K., Rosenthal I., Lounis N., Bishai W., Grosset J. (2005). Bactericidal activity of the nitroimidazopyran PA-824 in a murine model of tuberculosis. Antimicrob. Agents Chemother..

[95] Manjunatha U., Boshoff H.I., Barry C.E. (2009). The mechanism of action of PA-824: Novel insights from transcriptional profiling. Commun. Integr. Biol..

[96] Haver H.L., Chua A., Ghode P., Lakshminarayana S.B., Singhal A., Mathema B., Wintjens R., Bifani P. (2015). Mutations in genes for the F420 biosynthetic pathway and a nitroreductase enzyme are the primary resistance determinants in spontaneous in vitro-selected PA-824-resistant mutants of Mycobacterium tuberculosis. Antimicrob. Agents Chemother..

[97] Choi K.-P., Bair T.B., Bae Y.-M., Daniels L. (2001). Use of transposon Tn5367 mutagenesis and a nitroimidazopyran-based selection system to demonstrate a requirement for fbiA and fbiB in coenzyme F420 biosynthesis by Mycobacterium bovis BCG. J. Bacteriol..

[98] Conradie F., Diacon A.H., Ngubane N., Howell P., Everitt D., Crook A.M., Mendel C.M., Egizi E., Moreira J., Timm J. (2020). Treatment of highly drug-resistant pulmonary tuberculosis. N. Engl. J. Med..

[99] TB Alliance. https://www.tballiance.org/pathway-potential-new-tb-treatments.

[100] Keam S.J. (2019). Pretomanid: First Approval. Drugs..

[101] Deb U., Biswas S. (2020). Pretomanid: The latest USFDA-approved anti-tuberculosis drug. Indian J. Tuberc..

[102] Yang X., Wedajo W., Yamada Y., Dahlroth S.-L., Neo J.J.-L., Dick T., Chui W.K. (2018). 1, 3, 5-triazaspiro [5.5] undeca-2, 4-dienes as selective Mycobacterium tuberculosis dihydrofolate reductase inhibitors with potent whole cell activity. Eur. J. Med. Chem..

[103] Jing W., Zhang T., Zong Z., Xue Y., Shang Y., Wang F., Huang H., Chu N., Pang Y. (2019). Comparison of in vitro activity of the nitroimidazoles delamanid and pretomanid against multidrug-resistant and extensively drug-resistant tuberculosis. Eur. J. Clin. Microbiol. Infect. Dis..

[104] Jia L., Noker P.E., Coward L., Gorman G.S., Protopopova M., Tomaszewski J.E. (2006). Interspecies pharmacokinetics and in vitro metabolism of SQ109. Br. J. Pharmacol..

[105] Clain J.M., Escalante P. (2016). Novel treatments for drug-resistant tuberculosis. Clin. Med. Insights Ther..

[106] Dawson R., Diacon A.H., Everitt D., van Niekerk C., Donald P.R., Burger D.A., Schall R., Spigelman M., Conradie A., Eisenach K. (2015). Efficiency and safety of the combination of moxifloxacin, pretomanid (PA-824), and pyrazinamide during the first 8 weeks of antituberculosis treatment: A phase 2b, open-label, partly randomised trial in patients with drug-susceptible or drug-resistant pulmonary tuberculosis. Lancet.

[107] Diacon A.H., Dawson R., du Bois J., Narunsky K., Venter A., Donald P.R., van Niekerk C., Erondu N., Ginsberg A.M., Becker P. (2012). Phase II dose-ranging trial of the early bactericidal activity of PA-824. Antimicrob. Agents Chemother..

[108] Salinger D.H., Subramoney V., Everitt D., Nedelman J.R. (2019). Population pharmacokinetics of the antituberculosis agent pretomanid. Antimicrob. Agents Chemother..

[109] Dooley K.E., Luetkemeyer A.F., Park J.-G., Allen R., Cramer Y., Murray S., Sutherland D., Aweeka F., Koletar S.L., Marzan F. (2014). Phase I safety, pharmacokinetics, and pharmacogenetics study of the antituberculosis drug PA-824 with concomitant lopinavir-ritonavir, efavirenz, or rifampin. Antimicrob. Agents Chemother..

[110] Xu W.-C., Silverman M.H., Yu X.Y., Wright G., Brown N. (2019). Discovery and development of DNA polymerase IIIC inhibitors to treat Gram-positive infections. Bioorg. Med. Chem..

[111] Ginsberg A.M. (2010). Drugs in Development for Tuberculosis. Drugs..

[112] Conradie F., Diacon A., Everitt D., Mendel C., van Niekerk C., Howell P. Sustained high rate of successful treatment outcomes: Interim results of 75 patients in the Nix-TB clinical study of pretomanid, bedaquiline and linezolid. Proceedings of the 49th World Conference on Lung Health of the International Union Against Tuberculosis and Lung Disease.

[113] Kmentova I., Sutherland H.S., Palmer B.D., Blaser A., Franzblau S.G., Wan B., Wang Y., Ma Z., Denny W.A., Thompson A.M. (2010). Synthesis and structure− activity relationships of aza-and diazabiphenyl analogues of the antitubercular drug (6 S)-2-Nitro-6-{[4-(trifluoromethoxy) benzyl] oxy}-6, 7-dihydro-5 H-imidazo [2, 1-b][1, 3] oxazine (PA-824). J. Med. Chem..

[114] Upton A., Cho S., Yang T., Kim Y., Wang Y., Lu Y., Wang B., Xu J., Mdluli K., Ma Z. (2015). In vitro and in vivo activities of the nitroimidazole TBA-354 against Mycobacterium tuberculosis. Antimicrob. Agents Chemother..

[115] Da Silva P.B., Campos D.L., Ribeiro C.M., da Silva I.C., Pavan F.R. (2017). New antimycobacterial agents in the pre-clinical phase or beyond: Recent advances in patent literature (2001–2016). Expert Opin. Pat..

[116] Wallis R.S., Maeurer M., Mwaba P., Chakaya J., Rustomjee R., Migliori G.B., Marais B., Schito M., Churchyard G., Swaminathan S. (2016). Tuberculosis—advances in development of new drugs, treatment regimens, host-directed therapies, and biomarkers. Lancet Infect. Dis..

[117] TB Alliance. https://www.newtbdrugs.org/pipeline/compound/tba-354.

[118] Ntshangase S., Shobo A., Kruger H.G., Asperger A., Niemeyer D., Arvidsson P.I., Govender T., Baijnath S. (2018). The downfall of TBA-354-a possible explanation for its neurotoxicity via mass spectrometric imaging. Xenobiotica.

[119] Pandit N., Singla R.K., Shrivastava B. (2012). Current updates on oxazolidinone and its significance. Int. J. Med. Chem..

[120] Marchese A., Schito G. (2001). The oxazolidinones as a new family of antimicrobial agent. Clin. Microbiol. Infect..

[121] Kanafani Z.A., Corey G.R. (2012). Tedizolid (TR-701): A new oxazolidinone with enhanced potency. Expert Opin. Investig. Drugs.

[122] Carena A.A., Stryjewski M.E. (2020). Tedizolid (torezolid) for the treatment of complicated skin and skin structure infections. Expert Rev. Clin. Pharmacol..

[123] Shorr A.F., Lodise T.P., Corey G.R., De Anda C., Fang E., Das A.F., Prokocimer P. (2015). Analysis of the phase 3 establish trials of tedizolid versus linezolid in acute bacterial skin and skin structure infections. Antimicrob. Agents Chemother..

[124] Alcalá L., Ruiz-Serrano M.J., Turégano C.P.-F., de Viedma D.G., Díaz-Infantes M., Marín-Arriaza M., Bouza E. (2003). In vitro activities of linezolid against clinical isolates of Mycobacterium tuberculosis that are susceptible or resistant to first-line antituberculous drugs. Antimicrob. Agents Chemother..

[125] Stalker D.J., Jungbluth G.L., Hopkins N.K., Batts D.H. (2003). Pharmacokinetics and tolerance of single-and multiple-dose oral or intravenous linezolid, an oxazolidinone antibiotic, in healthy volunteers. J. Antimicrob. Chemother..

[126] Tang S., Yao L., Hao X., Zhang X., Liu G., Liu X., Wu M., Zen L., Sun H., Liu Y. (2015). Efficacy, safety and tolerability of linezolid for the treatment of XDR-TB: A study in China. Eur. Respir. J..

[127] Tsuji B.T., Kaatz G., Rybak M., Yu L., Edwards G., McKinnon P.S., Peloquin C., Morse G.D. (2005). Linezolid and other oxazolidinones. Antimicrobial Therapy and Vaccines.

[128] Diacon A.H., De Jager V.R., Dawson R., Narunsky K., Vanker N., Burger D.A., Everitt D., Pappas F., Nedelman J., Mendel C.M. (2020). Fourteen-day bactericidal activity, safety, and pharmacokinetics of linezolid in adults with drug-sensitive pulmonary tuberculosis. Antimicrob. Agents Chemother..

[129] Williams K.N., Brickner S.J., Stover C.K., Zhu T., Ogden A., Tasneen R., Tyagi S., Grosset J.H., Nuermberger E.L. (2009). Addition of PNU-100480 to first-line drugs shortens the time needed to cure murine tuberculosis. Am. J. Respir. Crit. Care Med..

[130] Gualano G., Capone S., Matteelli A., Palmieri F. (2016). New antituberculosis drugs: From clinical trial to programmatic use. Curr. Infect. Dis. Rep..

[131] Wallis R.S., Dawson R., Friedrich S.O., Venter A., Paige D., Zhu T., Silvia A., Gobey J., Ellery C., Zhang Y. (2014). Mycobactericidal activity of sutezolid (PNU-100480) in sputum (EBA) and blood (WBA) of patients with pulmonary tuberculosis. PLoS ONE.

[132] Dooley K.E., Kim P.S., Williams S.D., Hafner R. (2012). TB and HIV therapeutics: Pharmacology research priorities. AIDS Res Treat.

[133] Louie A., Eichas K., Files K., Swift M., Bahniuk N., Brown D., Drusano G.L. Activities of PNU-100480 (PNU 480) alone, PNU 480 plus its major metabolite PNU-101603 (PNU 1603) and PNU 480 plus PNU 1603 in combination with rifampin (RIF) against Mycobacterium tuberculosis: Comparison with linezolid. Proceedings of the 51st Interscience Conference on Antimicrobial Agents and Chemotherapy.

[134] TB Alliance. https://www.tballiance.org/news/tb-alliance-sublicenses-promising-anti-tuberculosis-drug-medicines-patent-pool.

[135] Libardo M.D., Boshoff H.I., Barry C.E. (2018). The present state of the tuberculosis drug development pipeline. Curr. Opin. Pharmacol..

[136] Wallis R.S., Jakubiec W., Kumar V., Bedarida G., Silvia A., Paige D., Zhu T., Mitton-Fry M., Ladutko L., Campbell S. (2011). Biomarker-assisted dose selection for safety and efficacy in early development of PNU-100480 for tuberculosis. Antimicrob. Agents Chemother..

[137] Zhu T., Friedrich S.O., Diacon A., Wallis R.S. (2014). Population pharmacokinetic/pharmacodynamic analysis of the bactericidal activities of sutezolid (PNU-100480) and its major metabolite against intracellular Mycobacterium tuberculosis in ex vivo whole-blood cultures of patients with pulmonary tuberculosis. Antimicrob. Agents Chemother..

[138] Furin J.J., Du Bois J., van Brakel E., Chheng P., Venter A., Peloquin C.A., Alsultan A., Thiel B.A., Debanne S.M., Boom W.H. (2016). Early bactericidal activity of AZD5847 in patients with pulmonary tuberculosis. Antimicrob. Agents Chemother..

[139] Zhang M., Sala C., Dhar N., Vocat A., Sambandamurthy V.K., Sharma S., Marriner G., Balasubramanian V., Cole S.T. (2014). In vitro and in vivo activities of three oxazolidinones against nonreplicating Mycobacterium tuberculosis. Antimicrob. Agents Chemother..

[140] Mistry N., Tolani M., Dholakia Y. (2015). New drugs for tuberculosis. Drugs Future..

[141] Balasubramanian V., Solapure S., Shandil R., Gaonkar S., Mahesh K., Reddy J., Deshpande A., Bharath S., Kumar N., Wright L. (2014). Pharmacokinetic and pharmacodynamic evaluation of AZD5847 in a mouse model of tuberculosis. Antimicrob. Agents Chemother..

[142] Kisgen J.J., Mansour H., Unger N.R., Childs L.M. (2014). Tedizolid: A new oxazolidinone antimicrobial. Am. J. Health Syst Pharm..

[143] Zhanel G.G., Love R., Adam H., Golden A., Zelenitsky S., Schweizer F., Gorityala B., Lagacé-Wiens P.R., Rubinstein E., Walkty A. (2015). Tedizolid: A novel oxazolidinone with potent activity against multidrug-resistant gram-positive pathogens. Drugs.

[144] Ong V., Flanagan S., Fang E., Dreskin H.J., Locke J.B., Bartizal K., Prokocimer P. (2014). Absorption, Distribution, Metabolism, and Excretion of the novel antibacterial prodrug tedizolid phosphate. Drug Metab. Dispos..

[145] Flanagan S., McKee E.E., Das D., Tulkens P.M., Hosako H., Fiedler-Kelly J., Passarell J., Radovsky A., Prokocimer P. (2015). Nonclinical and pharmacokinetic assessments to evaluate the potential of tedizolid and linezolid to affect mitochondrial function. Antimicrob. Agents Chemother..

[146] Fugitt R.B., Luckenbaugh R.W. (1982). 3-(p-Alkylsulfonylphenyl) Oxazolidinone Derivatives as Antibacterial Agents. U.S. Patent.

[147] Michalska K., Karpiuk I., Król M., Tyski S. (2013). Recent development of potent analogues of oxazolidinone antibacterial agents. Bioorg. Med. Chem..

[148] Vera-Cabrera L., Castro-Garza J., Rendon A., Ocampo-Candiani J., Welsh O., Choi S.H., Blackwood K., Molina-Torres C. (2005). In vitro susceptibility of mycobacterium tuberculosis clinical isolates to garenoxacin and DA-7867. Antimicrob. Agents Chemother..

[149] Ruiz P., Causse M., Vaquero M., Casal M. (2019). In vitro activity of tedizolid against Mycobacterium tuberculosis. Antimicrob. Agents Chemother..

[150] Deshpande D., Srivastava S., Nuermberger E., Koeuth T., Martin K.R., Cirrincione K.N., Lee P.S., Gumbo T. (2018). Multiparameter responses to tedizolid monotherapy and moxifloxacin combination therapy models of children with intracellular tuberculosis. Clin. Infect. Dis..

[151] Srivastava S., van Rijn S.P., Wessels A.M.A., Alffenaar J.-W.C., Gumbo T. (2016). Susceptibility testing of antibiotics that degrade faster than the doubling time of slow-growing mycobacteria: Ertapenem sterilizing effect versus Mycobacterium tuberculosis. Antimicrob. Agents Chemother..

[152] Hoffner S., Chan M.M., Chan E.D., Ordway D., Kesharwani P., Chopra S., Dasgupta A. (2020). Drug discovery targeting drug-resistant nontuberculous mycobacteria. Drug Discovery Targeting Drug-Resistant Bacteria, 4.

[153] Kim Y., Kim A., Lee S., Choi S.-H., Lee D.Y., Song J.-S., Lee H., Jang I.J., Yu K.S. (2017). Pharmacokinetics, safety, and tolerability of tedizolid phosphate after single-dose administration in healthy Korean male subjects. Clin. Ther..

[154] Eum S.Y., Kong J.H., Hong M.S., Lee Y.J., Kim J.H., Hwang S.H., Cho S.N., Via L.E., Barry C.E. (2010). Neutrophils are the predominant infected phagocytic cells in the airways of patients with active pulmonary TB. Chest.

[155] Srivastava S., Deshpande D., Nuermberger E., Lee P.S., Cirrincione K., Dheda K., Gumbo T. (2018). The Sterilizing effect of intermittent tedizolid for pulmonary tuberculosis. Clin. Infect. Dis..

[156] Sacksteder K.A., Protopopova M., Barry C.E., Andries K., Nacy C.A. (2012). Discovery and development of SQ109: A new antitubercular drug with a novel mechanism of action. Future Microbiol..

[157] Lee R.E., Protopopova M., Crooks E., Slayden R.A., Terrot M., Barry C.E. (2003). Combinatorial lead optimization of [1, 2]-diamines based on ethambutol as potential antituberculosis preclinical candidates. J. Comb. Chem..

[158] Su C.-C., Klenotic P.A., Bolla J.R., Purdy G.E., Robinson C.V., Yu E.W. (2019). MmpL3 is a lipid transporter that binds trehalose monomycolate and phosphatidylethanolamine. Proc. Natl. Acad. Sci. USA.

[159] Heinrich N., Dawson R., du Bois J., Narunsky K., Horwith G., Phipps A.J., Nacy C.A., Aarnoutse R.E., Boeree M.J., Gillespie S.H. (2015). Early phase evaluation of SQ109 alone and in combination with rifampicin in pulmonary TB patients. J. Antimicrob. Chemother..

[160] Protopopova M., Hanrahan C., Nikonenko B., Samala R., Chen P., Gearhart J., Einck L., Nacy C.A. (2005). Identification of a new antitubercular drug candidate, SQ109, from a combinatorial library of 1, 2-ethylenediamines. J. Antimicrob. Chemother..

[161] Nikonenko B.V., Protopopova M., Samala R., Einck L., Nacy C.A. (2007). Drug therapy of experimental tuberculosis (TB): Improved outcome by combining SQ109, a new diamine antibiotic, with existing TB drugs. Antimicrob. Agents Chemother..

[162] Boeree M.J., Heinrich N., Aarnoutse R., Diacon A.H., Dawson R., Rehal S., Kibiki G.S., Churchyard G., Sanne I., Ntinginya N.E. (2017). High-dose rifampicin, moxifloxacin, and SQ109 for treating tuberculosis: A multi-arm, multi-stage randomised controlled trial. Lancet Infect. Dis..

[163] Jia L., Tomaszewski J.E., Hanrahan C., Coward L., Noker P., Gorman G., Nikonenko B., Protopopova M. (2005). Pharmacodynamics and pharmacokinetics of SQ109, a new diamine-based antitubercular drug. Br. J. Pharmacol..

[164] Niemi M., Backman J.T., Fromm M.F., Neuvonen P.J., Kivistö K.T. (2003). Pharmacokinetic interactions with rifampicin. Clin. Pharmacokinet..

[165] Vilchèze C. (2020). Mycobacterial Cell Wall: A Source of Successful Targets for Old and New Drugs. Appl. Sci..

[166] Working Group for New TB Drugs. https://www.newtbdrugs.org/pipeline/trials.

[167] Kang S., Kim Y.M., Jeon H., Park S., Seo M.J., Lee S., Park D., Nam J., Lee S., Nam K. (2017). Synthesis and structure-activity relationships of novel fused ring analogues of Q203 as antitubercular agents. Eur. J. Med. Chem..

[168] Lohrasbi V., Talebi M., Bialvaei A.Z., Fattorini L., Drancourt M., Heidary M., Darban-Sarokhalil D. (2018). Trends in the discovery of new drugs for Mycobacterium tuberculosis therapy with a glance at resistance. Tuberculosis.

[169] Matsoso L.G., Kana B.D., Crellin P.K., Lea-Smith D.J., Pelosi A., Powell D., Powell D., Dawes S.S., Rubin H., Coppel R.L. (2005). Function of the cytochrome bc1-aa3 branch of the respiratory network in mycobacteria and network adaptation occurring in response to its disruption. J. Bacteriol..

[170] Kang S., Kim R.Y., Seo M.J., Lee S., Kim Y.M., Seo M., Seo J.J., Ko Y., Choi I., Jang J. (2014). Lead optimization of a novel series of imidazo [1, 2-a] pyridine amides leading to a clinical candidate (Q203) as a multi-and extensively-drug-resistant anti-tuberculosis agent. J. Med. Chem..

[171] Tang J., Wang B., Wu T., Wan J., Tu Z., Njire M., Wan B., Franzblauc S.G., Zhang T., Lu X. (2015). Design, synthesis, and biological evaluation of pyrazolo [1, 5-a] pyridine-3-carboxamides as novel antitubercular agents. ACS Medicinal Chem. Lett..

[172] Lu X., Williams Z., Hards K., Tang J., Cheung C.-Y., Aung H.L., Wang B., Liu Z., Hu X., Lenaerts A. (2018). Pyrazolo [1, 5-a] pyridine inhibitor of the respiratory cytochrome bcc complex for the treatment of drug-resistant tuberculosis. ACS Infect. Dis..

[173] Yu W., Chiwala G., Gao Y., Liu Z., Sapkota S., Lu Z., Guo L., Khan S.A., Zhong N., Zhang T. (2020). TB47 and clofazimine form a highly synergistic sterilizing block in a second-line regimen for tuberculosis in mice. Biomed. Pharmacother..

[174] Lu X., Tang J., Liu Z., Li M., Zhang T., Zhang X., Ding K. (2016). Discovery of new chemical entities as potential leads against Mycobacterium tuberculosis. Bioorg. Med. Chem. Lett..

[175] Grosset J.H., Tyagi S., Almeida D.V., Converse P.J., Li S.-Y., Ammerman N.C., Bishai W.R., Enarson D., Trébucq A. (2013). Assessment of clofazimine activity in a second-line regimen for tuberculosis in mice. Am. J. Respir. Crit. Care Med..

[176] Moraski G.C., Seeger N., Miller P.A., Oliver A.G., Boshoff H.I., Cho S., Mulugeta S., Anderson J.R., Franzblau S.G., Miller M.J. (2016). Arrival of imidazo [2, 1-b] thiazole-5-carboxamides: Potent anti-tuberculosis agents that target QcrB. ACS Infect. Dis..

[177] Moraski G.C., Deboosère N., Marshall K.L., Weaver H.A., Vandeputte A., Hastings C., Woolhiser L., Lenaerts A.J., Brodin P., Miller M.J. (2020). Intracellular and in vivo evaluation of imidazo [2, 1-b] thiazole-5-carboxamide anti-tuberculosis compounds. PLoS ONE.

[178] Trefzer C., Škovierová H., Buroni S., Bobovská A., Nenci S., Molteni E., Pojer F., Pasca M.R., Makarov V., Cole S.T. (2012). Benzothiazinones are suicide inhibitors of mycobacterial decaprenylphosphoryl-β-d-ribofuranose 2′-oxidase DprE1. J. Am. Chem. Soc..

[179] Shetye G.S., Franzblau S.G. (2020). New tuberculosis drug targets, their inhibitors and potential therapeutic impact. Transl. Res..

[180] Lechartier B., Hartkoorn R.C., Cole S.T. (2012). In vitro combination studies of benzothiazinone lead compound BTZ043 against Mycobacterium tuberculosis. Antimicrob. Agents Chemother..

[181] Makarov V., Manina G., Mikusova K., Möllmann U., Ryabova O., Saint-Joanis B., Dhar N., Pasca M.R., Buroni S., Lucarelli A.P. (2009). Benzothiazinones kill Mycobacterium tuberculosis by blocking arabinan synthesis. Science.

[182] Gao C., Peng C., Shi Y., You X., Ran K., Xiong L., Ye T.H., Zhang L., Wang N., Zhu Y. (2016). Benzothiazinethione is a potent preclinical candidate for the treatment of drug-resistant tuberculosis. Sci. Rep..

[183] Mahapatra S., Basu J., Brennan P., Crick D., Cole S., Eisenach K., McMurray D.N., Jacobs W.R. (2005). Tuberculosis and the Tubercle Bacillus. Emerg. Infect. Dis..

[184] Makarov V., Lechartier B., Zhang M., Neres J., van der Sar A.M., Raadsen S.A., Hartkoorn R.C., Ryabova O.B., Vocat A., Decosterd L.A. (2014). Towards a new combination therapy for tuberculosis with next generation benzothiazinones. EMBO Mol. Med..

[185] Makarov V., Mikušová K. (2020). Development of macozinone for TB treatment: An update. Appl. Sci..

[186] Shi J., Lu J., Zong Z., Huo F., Luo J., Liang Q., Li Y., Huang H., Pang Y. (2018). In vitro activity of PBTZ169 against multiple Mycobacterium species. Antimicrob. Agents Chemother..

[187] Lechartier B., Cole S.T. (2015). Mode of action of clofazimine and combination therapy with benzothiazinones against Mycobacterium tuberculosis. Antimicrob. Agents Chemother..

[188] Mariandyshev A., Khokhlov A., Smerdin S., Shcherbakova V., Igumnova O., Ozerova I., Bolgarina A.A., Nikitina N.A. (2020). The main results of clinical trials of the efficacy, safety and pharmacokinetics of the perspective anti-tuberculosis drug makozinone (PBTZ169). Ter. Arkh..

[189] Spaggiari D., Desfontaine V., Cruchon S., Guinchard S., Vocat A., Blattes E., Pitteloud J., Ciullini L., Bardinet C., Ivanyuk A. (2019). Development and validation of a multiplex UHPLC-MS/MS method for the determination of the investigational antibiotic against multi-resistant tuberculosis macozinone (PBTZ169) and five active metabolites in human plasma. PLoS ONE.

[190] Kloss F., Krchnak V., Krchnakova A., Schieferdecker S., Dreisbach J., Krone V., Möllmann U., Hoelscher M., Miller M.J. (2017). In vivo dearomatization of the potent antituberculosis agent BTZ043 via Meisenheimer complex formation. Angew. Chem. Int..

[191] Yuan T., Sampson N.S. (2018). Hit generation in TB drug discovery: From genome to granuloma. Chem. Rev..

[192] Torfs E., Piller T., Cos P., Cappoen D. (2019). Opportunities for overcoming Mycobacterium tuberculosis drug resistance: Emerging mycobacterial targets and host-directed therapy. Int. J. Mol. Sci..

[193] Riccardi G., Pasca M.R., Chiarelli L.R., Manina G., Mattevi A., Binda C. (2013). The DprE1 enzyme, one of the most vulnerable targets of Mycobacterium tuberculosis. Appl. Microbiol. Biotechnol..

[194] Panda M., Ramachandran S., Ramachandran V., Shirude P.S., Humnabadkar V., Nagalapur K., Sharma S., Kaur P., Guptha S., Narayan A. (2014). Discovery of pyrazolopyridones as a novel class of noncovalent DprE1 inhibitor with potent anti-mycobacterial activity. J. Med. Chem..

[195] Hariguchi N., Chen X., Hayashi Y., Kawano Y., Fujiwara M., Matsuba M., Shimizu H., Ohba Y., Nakamura I., Kitamoto R. (2020). OPC-167832, a novel carbostyril derivative with potent anti-tuberculosis activity as a DprE1 inhibitor. Antimicrob. Agents Chemother..

[196] Milano A., Pasca M.R., Provvedi R., Lucarelli A.P., Manina G., Ribeiro A.L.dJ.L., Manganelli R., Riccardi G. (2009). Azole resistance in Mycobacterium tuberculosis is mediated by the MmpS5–MmpL5 efflux system. Tuberculosis.

[197] Heifets L.B. (1994). Antimycobacterial drugs. Semin Respir. Infect..

[198] Shirude P.S., Shandil R., Sadler C., Naik M., Hosagrahara V., Hameed S., Shinde V., Bathula C., Humnabadkar V., Kumar N. (2013). Azaindoles: Noncovalent DprE1 inhibitors from scaffold morphing efforts, kill Mycobacterium tuberculosis and are efficacious in vivo. J. Med. Chem..

[199] Chatterji M., Shandil R., Manjunatha M., Solapure S., Ramachandran V., Kumar N., Saralaya R., Panduga V., Reddy J., Prabhakar K.R. (2014). 1,4-Azaindole, a potential drug candidate for treatment of tuberculosis. Antimicrob. Agents Chemother..

[200] Shirude P.S., Shandil R.K., Manjunatha M., Sadler C., Panda M., Panduga V., Reddy J., Saralaya R., Nanduri R., Ambady A. (2014). Lead optimization of 1,4-azaindoles as antimycobacterial agents. J. Med. Chem..

[201] Igarashi M., Nakagawa N., Doi N., Hattori S., Naganawa H., Hamada M. (2003). Caprazamycin B, a novel anti-tuberculosis antibiotic, from *Streptomyces* sp.. J. Antibiot. Res..

[202] Hirano S., Ichikawa S., Matsuda A. (2008). Structure–activity relationship of truncated analogs of caprazamycins as potential anti-tuberculosis agents. Bioorg. Med. Chem..

[203] Wiker F., Hauck N., Grond S., Gust B. (2019). Caprazamycins: Biosynthesis and structure activity relationship studies. Int. J. Med. Microbiol..

[204] Bugg T.D., Lloyd A.J., Roper D.I. (2006). Phospho-MurNAc-pentapeptide translocase (MraY) as a target for antibacterial agents and antibacterial proteins. Infect. Disord. Drug Targets.

[205] Nakamura H., Yoshida T., Tsukano C., Takemoto Y. (2016). Synthesis of CPZEN-45: Construction of the 1, 4-diazepin-2-one core by the Cu-catalyzed intramolecular amidation of a vinyl iodide. Org. Lett..

[206] Ishizaki Y., Hayashi C., Inoue K., Igarashi M., Takahashi Y., Pujari V., Crick D.C., Brennan P.J., Nomoto A. (2013). Inhibition of the first step in synthesis of the mycobacterial cell wall core, catalyzed by the GlcNAc-1-phosphate transferase WecA, by the novel caprazamycin derivative CPZEN-45. J. Biol. Chem..

[207] Takahashi Y., Igarashi M., Miyake T., Soutome H., Ishikawa K., Komatsuki Y., Koyama Y., Nakagawa N., Hattori S., Inoue K. (2013). Novel semisynthetic antibiotics from caprazamycins A–G: Caprazene derivatives and their antibacterial activity. J. Antibiot. Res..

[208] Pitner R.A., Durham P.G., Stewart I.E., Reed S.G., Cassell G.H., Hickey A.J., Carter D. (2019). A Spray-dried combination of capreomycin and CPZEN-45 for inhaled tuberculosis therapy. J. Pharm. Sci..

[209] Salomon J.J., Galeron P., Schulte N., Morow P.R., Severynse-Stevens D., Huwer H., Daum N., Lehr C.M., Hickey A.J., Ehrhardt C. (2013). Biopharmaceutical in vitro characterization of CPZEN-45, a drug candidate for inhalation therapy of tuberculosis. Ther. Deliv..

[210] Barry V.C., Belton J.G., Conalty M.L., Twomey D. (1948). Anti-tubercular activity of oxidation products of substituted o-phenylene diamines. Nature.

[211] Barry V.C., Buggle K., Byrne J., Conalty M.L., Winder F. (1960). Absorption, distribution and retention of the rimino-compounds in the experimental animal. Ir. J. Med. Sci..

[212] Reddy V.M., O’ Sullivan J.F., Gangadharam P.R.J. (1999). Antimycobacterial activities of riminophenazines. J. Antimicrob. Chemother..

[213] Bvumbi M.V. (2020). Activity of riminophenazines against mycobacterium tuberculosis: A Review of Studies that Might be Contenders for Use as Antituberculosis Agents. ChemMedChem.

[214] Vischer W.A. (1969). The experimental properties of G 30 320 (B 663)—A new anti-leprotic agent. Lepr. Rev..

[215] Barry V.C., Conalty M.L. (1958). Antituberculosis activity in the phenazine series: II. N3-substituted analinooposafranmes (rimino-compounds) and some derivatives. Int. J. Tuberc. Lung Dis..

[216] Browne S., Hogerzeil L. (1962). “B 663” in the treatment of leprosy. Preliminary report of a pilot trial. Lepr. Rev..

[217] Barry V.C., Belton J.G., Conalty M.L., Denneny J.M., Edward D.W., O’sullivan J.F., Twomey D., Winder F. (1957). A new series of phenazines (rimino-compounds) with high antituberculosis activity. Nature.

[218] Yano T., Kassovska-Bratinova S., Teh J.S., Winkler J., Sullivan K., Isaacs A., Schechter N.M., Rubin H. (2011). Reduction of clofazimine by mycobacterial type 2 NADH: Quinone oxidoreductase a pathway for the generation of bactericidal levels of reactive oxygen species. J. Biol. Chem..

[219] Yawalkar S., Vischer W. (1979). Lamprene (clofazimine) in leprosy. Lepr. Rev..

[220] Xu J., Wang B., Fu L., Zhu H., Guo S., Huang H., Yin D., Zhang Y., Lu Y. (2019). In vitro and in vivo activities of the riminophenazine TBI-166 against Mycobacterium tuberculosis. Antimicrob. Agents Chemother..

[221] Li D., Sheng L., Liu X., Yang S., Liu Z., Li Y. (2014). Determination of TBI-166, a novel antituberculotic, in Rat plasma by liquid chromatography–tandem mass spectrometry. Chromatographia.

[222] Lu Y., Zheng M., Wang B., Fu L., Zhao W., Li P., Xu J., Zhu H., Jin H., Yin D. (2011). Clofazimine analogs with efficacy against experimental tuberculosis and reduced potential for accumulation. Antimicrob. Agents Chemother..

[223] Li Q., Lu X. (2020). New antituberculosis drugs targeting the respiratory chain. Chin. Chem. Lett..

[224] Mohamed M., El-Hameed A., Sayed A. (2017). Synthesis strategies and biological value of pyrrole and pyrrolopyrimidine. J. Adv. Pharm. Technol. Res..

[225] Castro A.J., Gale G.R., Means G.E., Tertzakian G. (1967). Antimicrobial properties of pyrrole derivatives. J. Med. Chem..

[226] Mohamed M.S., Fathallah S.S. (2014). Pyrroles and fused pyrroles: Synthesis and therapeutic activities. Mini. Rev. Org. Chem..

[227] Deidda D., Lampis G., Fioravanti R., Biava M., Porretta G.C., Zanetti S., Pompei R. (1998). Bactericidal activities of the pyrrole derivative BM212 against multidrug-resistant and intramacrophagic Mycobacterium tuberculosis strains. Antimicrob. Agents Chemother..

[228] La Rosa V., Poce G., Canseco J.O., Buroni S., Pasca M.R., Biava M., Raju R.M., Porretta G.C., Alfonso S., Battilocchio C. (2012). MmpL3 is the cellular target of the antitubercular pyrrole derivative BM212. Antimicrob. Agents Chemother..

[229] Xu Z., Meshcheryakov V.A., Poce G., Chng S.-S. (2017). MmpL3 is the flippase for mycolic acids in mycobacteria. Proc. Natl. Acad. Sci. USA.

[230] Bhakta S., Scalacci N., Maitra A., Brown A.K., Dasugari S., Evangelopoulos D., McHugh T.D., Mortazavi P.N., Twist A., Petricci E. (2016). Design and synthesis of 1-((1, 5-Bis (4-chlorophenyl)-2-methyl-1 H-pyrrol-3-yl) methyl)-4-methylpiperazine (BM212) and N-Adamantan-2-yl-N′-((E)-3, 7-dimethylocta-2, 6-dienyl) ethane-1, 2-diamine (SQ109) pyrrole hybrid derivatives: Discovery of potent antitubercular agents effective against multidrug-resistant mycobacteria. J. Med. Chem..

[231] Nikonenko B.V., Samala R., Einck L., Nacy C.A. (2004). Rapid, simple in vivo screen for new drugs active against Mycobacterium tuberculosis. Antimicrob. Agents Chemother..

[232] Mahboub B., Vats M. (2013). Tuberculosis: Current Issues in Diagnosis and Management..

[233] Adhvaryu M., Vakharia B. (2011). Drug-resistant tuberculosis: Emerging treatment options. Clin. Pharmacol..

[234] Van den Boogaard J., Kibiki G.S., Kisanga E.R., Boeree M.J., Aarnoutse R.E. (2009). New drugs against tuberculosis: Problems, progress, and evaluation of agents in clinical development. Antimicrob. Agents Chemother..

[235] Dessen A., Quemard A., Blanchard J.S., Jacobs W.R., Sacchettini J.C. (1995). Crystal structure and function of the isoniazid target of Mycobacterium tuberculosis. Science.

[236] Rawat R., Whitty A., Tonge P.J. (2003). The isoniazid-NAD adduct is a slow, tight-binding inhibitor of InhA, the Mycobacterium tuberculosis enoyl reductase: Adduct affinity and drug resistance. Proc. Natl. Acad. Sci. USA.

[237] Banerjee A., Dubnau E., Quemard A., Balasubramanian V., Um K.S., Wilson T., Collins D., De Lisle G., Jacobs W.R. (1994). inhA, a gene encoding a target for isoniazid and ethionamide in Mycobacterium tuberculosis. Science.

[238] Zhang Y., Heym B., Allen B., Young D., Cole S. (1992). The catalase—Peroxidase gene and isoniazid resistance of Mycobacterium tuberculosis. Nature.

[239] Shirude P.S., Madhavapeddi P., Naik M., Murugan K., Shinde V., Nandishaiah R., Bhat J., Kumar A., Hameed S., Holdgate G. (2013). Methyl-thiazoles: A novel mode of inhibition with the potential to develop novel inhibitors targeting InhA in Mycobacterium tuberculosis. J. Med. Chem..

[240] Kamsri P., Hanwarinroj C., Phusi N., Pornprom T., Chayajarus K., Punkvang A., Suttipanta N., Srimanote P., Suttisintong K., Songsiriritthigul C. (2019). Discovery of new and potent InhA inhibitors as anti-tuberculosis agents: Structure based virtual screening validated by biological assays and x-ray crystallography. J. Chem. Inf. Model..

[241] Martínez-Hoyos M., Perez-Herran E., Gulten G., Encinas L., Álvarez-Gómez D., Alvarez E., Ferrer-Bazaga S., García-Pérez A., Ortega F., Angulo-Barturen I. (2016). Antitubercular drugs for an old target: GSK693 as a promising InhA direct inhibitor. EBioMedicine.

[242] He X., Alian A., Stroud R., Ortiz de Montellano P.R. (2006). Pyrrolidine carboxamides as a novel class of inhibitors of enoyl acyl carrier protein reductase from Mycobacterium tuberculosis. J. Med. Chem..

[243] Vasilyev D., Merrill T., Iwanow A., Dunlop J., Bowlby M. (2006). A novel method for patch-clamp automation. Pflügers Arch..

[244] Moulkrere B.R., Orena B.S., Mori G., Saffon-Merceron N., Rodriguez F., Lherbet C., Belkheiri N., Amari M., Hoffmann P., Fodili M. (2018). Evaluation of heteroatom-rich derivatives as antitubercular agents with InhA inhibition properties. Med. Chem Res..

[245] Barry C.E., Lee R.E., Mdluli K., Sampson A.E., Schroeder B.G., Slayden R.A., Yuan Y. (1998). Mycolic acids: Structure, biosynthesis and physiological functions. Prog. Lipid Res..

[246] Vilchèze C., Morbidoni H.R., Weisbrod T.R., Iwamoto H., Kuo M., Sacchettini J.C., Jacobs W.R. (2000). Inactivation of the inhA-encoded fatty acid synthase II (FASII) enoyl-acyl carrier protein reductase induces accumulation of the FASI end products and cell lysis of Mycobacterium smegmatis. J. Bacteriol..

[247] Zhang N.-N., Liu Z.-Y., Liang J., Tang Y.-X., Qian L., Gao Y.-M., Zhang T.Y., Yan M. (2018). Design, synthesis, and biological evaluation of m-amidophenol derivatives as a new class of antitubercular agents. MedChemComm.

[248] Flint L., Korkegian A., Parish T. (2020). InhA inhibitors have activity against non-replicating Mycobacterium. PLoS ONE.

[249] Bonnett S.A., Dennison D., Files M., Bajpai A., Parish T. (2018). A class of hydrazones are active against non-replicating Mycobacterium tuberculosis. PLoS ONE.

[250] Betts J.C., Lukey P.T., Robb L.C., McAdam R.A., Duncan K. (2002). Evaluation of a nutrient starvation model of Mycobacterium tuberculosis persistence by gene and protein expression profiling. Mol. Microbiol..

[251] McNeil M.B., Dennison D., Shelton C., Flint L., Korkegian A., Parish T. (2017). Mechanisms of resistance against NITD-916, a direct inhibitor of Mycobacterium tuberculosis InhA. Tuberculosis.

[252] Owens R.C. (2008). An overview of harms associated with beta-lactam antimicrobials: Where do the carbapenems fit in?. Crit. Care..

[253] Dhar N., Dubée V., Ballell L., Cuinet G., Hugonnet J.-E., Signorino-Gelo F., Barros D., Arthur M., McKinney J.D. (2015). Rapid cytolysis of Mycobacterium tuberculosis by faropenem, an orally bioavailable β-lactam antibiotic. Antimicrob. Agents Chemother..

[254] Milazzo I., Blandino G., Caccamo F., Musumeci R., Nicoletti G., Speciale A. (2003). Faropenem, a new oral penem: Antibacterial activity against selected anaerobic and fastidious periodontal isolates. J. Antimicrob. Chemother..

[255] Rullas J., Dhar N., McKinney J.D., García-Pérez A., Lelievre J., Diacon A.H., Hugonnet J.E., Arthur M., Angulo-Barturen I., Barros-Aguirre D. (2015). Combinations of β-lactam antibiotics currently in clinical trials are efficacious in a DHP-I-deficient mouse model of tuberculosis infection. Antimicrob. Agents Chemother..

[256] Tanaka E., Kimoto T., Tsuyuguchi K., Suzuki K., Amitani R. (2002). Successful treatment with faropenem and clarithromycin of pulmonary Mycobacterium abscessus infection. J. Infect. Chemother..

[257] Dubée V., Triboulet S., Mainardi J.-L., Ethève-Quelquejeu M., Gutmann L., Marie A., Dubost L., Hugonnet J.E., Arthur M. (2012). Inactivation of Mycobacterium tuberculosis L, D-transpeptidase LdtMt1 by carbapenems and cephalosporins. Antimicrob. Agents Chemother..

[258] Cordillot M., Dubée V., Triboulet S., Dubost L., Marie A., Hugonnet J.-E., Arthur M., Mainardi J.L. (2013). In vitro cross-linking of Mycobacterium tuberculosis peptidoglycan by L, D-transpeptidases and inactivation of these enzymes by carbapenems. Antimicrob. Agents Chemother..

[259] Van Rijn S.P., van Altena R., Akkerman O.W., van Soolingen D., van der Laan T., de Lange W.C., Kosterink J.G., van der Werf T.S., Alffenaar J.W. (2016). Pharmacokinetics of ertapenem in patients with multidrug-resistant tuberculosis. Eur. Respir. J..

[260] Musuka S., Srivastava S., Dona C.W.S., Meek C., Leff R., Pasipanodya J., Gumbo T. (2013). Thioridazine pharmacokinetic-pharmacodynamic parameters “wobble” during treatment of tuberculosis: A theoretical basis for shorter-duration curative monotherapy with congeners. Antimicrob. Agents Chemother..

[261] Teppler H., Gesser R.M., Friedland I.R., Woods G.L., Meibohm A., Herman G., Mistry G., Isaacs R. (2004). Safety and tolerability of ertapenem. J. Antimicrob. Chemother..

[262] Zhanel G.G., Wiebe R., Dilay L., Thomson K., Rubinstein E., Hoban D.J., Noreddin A.M., Karlowsky J.A. (2007). Comparative review of the carbapenems. Drugs.

[263] Van Rijn S.P., Srivastava S., Wessels M.A., van Soolingen D., Alffenaar J.-W.C., Gumbo T. (2017). Sterilizing effect of ertapenem-clavulanate in a hollow-fiber model of tuberculosis and implications on clinical dosing. Antimicrob. Agents Chemother..

[264] Tiberi S., D’Ambrosio L., De Lorenzo S., Viggiani P., Centis R., Sotgiu G., Alffenaar J.W., Migliori G.B. (2016). Ertapenem in the treatment of multidrug-resistant tuberculosis: First clinical experience. Eur. Respir. J..

[265] Nix D.E., Majumdar A.K., Di Nubile M.J. (2004). Pharmacokinetics and pharmacodynamics of ertapenem: An overview for clinicians. J. Antimicrob. Chemother..

[266] Shah P.M., Isaacs R.D. (2003). Ertapenem, the first of a new group of carbapenems. J. Antimicrob. Chemother..

[267] Keating G.M., Perry C.M. (2005). Ertapenem. Drugs.

[268] Lue S.W., Kelley S.O. (2005). An aminoacyl-tRNA synthetase with a defunct editing site. Biochemistry.

[269] Palencia A., Li X., Bu W., Choi W., Ding C.Z., Easom E.E., Feng L., Hernandez V., Houston P., Liu L. (2016). Discovery of novel oral protein synthesis inhibitors of Mycobacterium tuberculosis that target leucyl-tRNA synthetase. Antimicrob. Agents Chemother..

[270] World Health Organization. https://apps.who.int/iris/handle/10665/275487.

[271] Tenero D., Derimanov G., Carlton A., Tonkyn J., Davies M., Cozens S., Gresham S., Gaudion A., Puri A., Muliaditan M. (2019). First-time-in-human study and prediction of early bactericidal activity for GSK3036656, a potent leucyl-tRNA synthetase inhibitor for tuberculosis treatment. Antimicrob. Agents Chemother..

[272] Li X., Hernandez V., Rock F.L., Choi W., Mak Y.S., Mohan M., Mao W., Zhou Y., Easom E.E., Plattner J.J. (2017). Discovery of a potent and specific M. tuberculosis Leucyl-tRNA synthetase inhibitor:(S)-3-(Aminomethyl)-4-chloro-7-(2-hydroxyethoxy) benzo [c][1, 2] oxaborol-1 (3 H)-ol (GSK656). J. Med. Chem..

[273] Rullas J., García J.I., Beltrán M., Cardona P.-J., Cáceres N., García-Bustos J.F., Angulo-Barturen I. (2010). Fast standardized therapeutic-efficacy assay for drug discovery against tuberculosis. Antimicrob. Agents Chemother..

[274] Franzblau S.G., DeGroote M.A., Cho S.H., Andries K., Nuermberger E., Orme I.M., Mdluli K., Angulo-Barturen I., Dick T., Dartois V. (2012). Comprehensive analysis of methods used for the evaluation of compounds against Mycobacterium tuberculosis. Tuberculosis.

[275] Li S.-Y., Tasneen R., Tyagi S., Soni H., Converse P.J., Mdluli K., Nuermberger E.L. (2017). Bactericidal and sterilizing activity of a novel regimen with bedaquiline, pretomanid, moxifloxacin, and pyrazinamide in a murine model of tuberculosis. Antimicrob. Agents Chemother..

